# Comprehensive bioinformatic analysis of MMP1 in hepatocellular carcinoma and establishment of relevant prognostic model

**DOI:** 10.1038/s41598-022-17954-x

**Published:** 2022-08-10

**Authors:** Lei Dai, Joseph Mugaanyi, Xingchen Cai, Mingjun Dong, Caide Lu, Changjiang Lu

**Affiliations:** 1 Department of Hepatopancreatobiliary Surgery, Ningbo Medical Centre Lihuili Hospital, Ningbo University, 1111 Jiangnan Road, Ningbo, 315040 Zhejiang China; 2 Department of Emergency, Ningbo Medical Centre Lihuili Hospital, Ningbo University, Ningbo, 315040 Zhejiang China

**Keywords:** Cancer, Computational biology and bioinformatics, Genetics, Immunology, Molecular biology, Biomarkers, Oncology

## Abstract

Matrix metalloproteinase 1 (MMP1) encodes endopeptidases associated with degradation of multiple components of the extracellular matrix. This function has increasingly been considered to play a major proteolysis role in tumor invasion and metastasis. However, the relationship between MMP1 gene expression, tumor-immune microenvironment and prognosis in hepatocellular carcinoma patients remains mostly unclear. This study focused on a comprehensive analysis of MMP1 in hepatocellular carcinoma, specifically the prognosis and tumor-immune microenvironment. MMP1 expression was analyzed using TCGA database and clinical samples. MMP1 associated mechanisms, pathways, mutations and prognosis in hepatocellular carcinoma were evaluated. We also analyzed the tumor-immune microenvironment and corresponding treatments. Our research demonstrated that MMP1 expression was upregulated in patients with hepatocellular carcinoma and correlated with poor survival. A prognostic model was established and its performance evaluated. We also found and report various correlations between MMP1 and immune-related cells/genes, as well the potential therapeutic agents. These findings indicate that MMP1 can potentially be a promising prognostic biomarker and indicator of the tumor-immune microenvironment status in hepatocellular carcinoma.

## Introduction

Hepatocellular carcinoma (HCC), remains the third leading cause of tumor-related death worldwide^1^. Chronic hepatitis B-induced cirrhosis leading to HCC is the most common progress pattern in liver cancer. The other top three risk factors for HCC are alcohol consumption, chronic hepatitis C and non-alcoholic fatty liver disease^2^. Although great strides have been made in advancing early diagnosis, surgical technology^3^, targeted treatment^4,5^ and immunotherapy^6,7^, the high rate of recurrence and mortality remain a challenge. This is because most patients present with unresectable lesions or distant metastasis at time diagnosis. In such cases, prognosis is poor. The 5-year overall survival is only 10–18%^8–10^. Therefore, a novo prognosis prediction model is needed to aid in patient evaluation, treatment optimization and possibly improve patient outcome.

Matrix metalloproteinases (MMPs), a family of zinc-dependent endoproteases, is significantly associated with extracellular matrix degradation through protein denaturation, which plays a vital role in apoptosis, angiogenesis, and immune response^11–14^ of the tumor microenvironment. MMP-2 together with MMP-9, are the two most common progression markers correlated with invasion and metastasis in various tumor, especially HCC^15,16^. Although MMP1 has been more commonly reported to be expressed in non-neoplastic liver tissues^17^, it has also been shown to be associated with invasion and migration in HCC by extracellular matrix (ECM) degradation in the epithelial-mesenchymal transition (EMT)^18^. MMP1 can be expressed with a low positive rate under normal conditions in a wide range of cells including stromal fibroblasts, macrophages, endothelial cells and epithelial cells. However, it’s expression can be elevated in malignant tumors with poor prognosis (such as ovarian, liver, lung, gastric, colorectal, and prostate)^19–24^. While some studies have reported a relationship between MMP1 and HCC, its specific role in prognosis and the associated tumor-immunity are still unclear.

Furthermore, tumor infiltration immune cells (TIICs) and tumor-associated immune microenvironment are currently key areas of interest for researchers^25,26^. Immune-related cells and genes may respond to the tumor progression and metastasis through a multitude of pathways and interactions in HCC. The suppression of HCC immune microenvironment facilitates immune tolerance and escape through a number mechanisms^27^. MMPs play an important role in promoting bladder cancer metastasis through the B cell induced signaling pathway^28^ and their upregulation by tumor-associated macrophages contributes to tumor infiltration and metastasis in various carcinomas^29–31^, indicating their potential involvement in the tumor-immune microenvironment. However, which and how MMP1 influences the immune cells and the underlying microenvironment still needs to be explored.

In this study, we carried out a comprehensive investigation of the prognostic potential of MMP1 and its relationship with immune-related cells and genes in HCC.

## Materials and methods

### Genome structure analysis and overview of the mechanisms

We obtained the genome annotations of the MMP1 gene from the University of California Santa Cruz (UCSC) genome browser (http://genome.ucsc.edu/) on Human Dec 2013 (GRCh38/hg28) assembly^32^.

After searching through the relevant literature, we summarized and outlined the pathologic pathways and mechanisms mediated by MMP1 in different disorders and cancers based on present cell- or animal-experimental evidence.

### Gene expression analysis

The tumor immune estimation resource (TIMER), version 2.0 database (http://timer.comp-genomics.org), incorporating 10,009 samples across 23 cancer types from TCGA, is a comprehensive web resource for the systematical evaluation of the differential gene correlation and clinical relevance of tumor-immune infiltrates analysis^33–35^. We selected the “Gene_DE” module to explore the difference in MMP1 expression level between tumor and adjacent normal tissues for an array of carcinomas or their sub-types.

We downloaded the liver hepatocellular carcinoma (LIHC) data from TCGA database and conducted the differential expression analysis of MMP1 based on 50 paired samples.

Gene expression profiling interactive analysis (GEPIA) database (http://gepia.cancer-pku.cn/index.html), a public web server for tumor and normal gene expression profiling and interactive analysis based on the data from Genotype-Tissue Expression (GTEx) and TCGA database^36^, was used to analyze the difference in MMP1 expression in unpaired samples and each pathologic stage of LIHC. The log2(Transcripts per million (TPM) + 1) for log-scale was used in the assessments.

We included two gene expression datasets related to HCC (GSE14520 (n = 488) and GSE25097 (n = 557)) to conduct differentia expression validation analysis of MMP1.

### Genetic alteration analysis

Utilizing the cBioPortal (^37–39^, which is an open platform supporting multidimensional cancer genomics data, we performed a visualized genetic alteration analysis of MMP1. We selected “Quick select: TCGA PanCancer Atlas Studies” on the home page and submitted query for “MMP1” genetic variation characteristics. Data containing alteration frequency, structural variant, mutation and copy number alteration (CNA) was extracted. Next, we obtained mutated site summary of MMP1 exhibited in the pattern chart and three-dimensional (3D) plot of protein structure via the “Mutations” module. We re-selected “Liver Hepatocellular Carcinoma (TCGA, PanCancer Atlas)” on the home page to conduct a survival analysis in overall survival (OS), disease free survival (DFS), progress free survival (PFS) and disease specific survival (DSS) with/without MMP1 gene variation via the “Comparison/Survival” module. Expression difference between wild type (WT) and mutated MMP1 was compared via “Gene_Mutation” module of TIMER 2.0.

### DNA methylation and gene enrichment analysis

MEXPRESS (^40,41^ provided openly visualized DNA methylation, expression and clinical data, as well as statistical analysis using the Pearson correlation coefficient and Benjamini–Hochberg methods. Using this tool, we performed DNA methylation analysis between MMP1 gene of numerous probes (e.g., cg25320665, cg14543953, etc.) and LIHC.

We used the STRING database (^42^, which contains data on functional proteins association networks, to construct a MMP1-related protein–protein interaction (PPI) network. The main parameters were set as follows: protein name (“MMP1”), organism (“Homo sapiens”), meaning of network edges (“evidence”), active interaction sources (“Experiments”), minimum required interaction sore [“low confidence (0.150)”] and max number of interactors to show [“1st shell: no more than 50 interactors”].

Through analysis of the differential expression of genes, we acquired data for MMP1-related/similar genes from DESeq2 platform^43^ (version 1.26.0) based on TCGA-LIHC using R (version 3.6.3^44^. Next, we screened out the genes with log2FoldChange (log2FC) > 2/ < -2 and *p* value < 0.05 to conduct Gene ontology (GO) enrichment and Kyoto encyclopedia of genes and genomes (KEGG) pathway analysis. The results were visualized using “ggplot2” (version 3.3.3) and “clusterProfiler” (version 3.14.3) packages in R^45^. Functional analysis combined with log2FC was performed via R’s “Goplot” package (version 1.0.2)^46^. In details, biological process (BP), cellular component (CC) and molecular function (MF) were output visualized cnetplots (node_label = T, colorEdge = T, circular = F) respectively.

### Clinical correlation and survival analysis

Clinical data of 374 cases was extracted from TCGA-LIHC and cleansed. Based on MMP1 expression, we divided the patients into two groups (low expression (0–50%) vs high expression (50–100%)). We conducted the clinical correlation analysis between MMP1 expression and numerous clinical indexes (e.g., vascular invasion, pathologic stage, tumor status, etc.). We also evaluated the baseline characteristics of patients from TGCA-LIHC. A logistics regression model of related characteristics was performed and Odds Ratio (OR) calculated.

We retrieved the corresponding prognostic data^47^ as supplements to conduct prognostic analysis of OS, DSS and progress free interval (PFI). The prognostic data of PFS was obtained from the Kaplan–Meier Plotter database^48^ (http://kmplot.com/analysis), assembling gene microarray and RNA-seq data from the gene expression omnibus (GEO)^49^, European genome-phenome archive (EGA)^50^ and TCGA public databases. We conducted a series of OS analyses of subgroups to identify the high-risk factors related to MMP1 expression and prognosis. All the Kaplan–Meier curves were obtained via R’s “survival/survminer” package, so were hazard ratios (HR) and confident intervals (CI).

We also performed the univariate and multivariate analysis for the OS, DSS and PFI of TCGA-LIHC by log-rank test or cox regression model.

### Establishment of MMP1-related prognostic model

Based on the data of TCGA-LIHC, we initially evaluated the diagnostic potential of MMP1 via receiver operating character (ROC) curve. According to the previous analysis, we established an MMP1-related nomogram prognostic model involving 6 clinical indicators (Tumor status, T stage, M stage, Pathologic stage, Age and Histologic grade) to predict 2–4 years OS probability in HCC patients. Next, we conducted time-dependent ROC curves, decision curve analysis (DCA) and prognostic calibration analysis to verify the reliability and accuracy of the model.

### Experimental validation of MMP1 expression

We conducted Western blotting (WB) and real-time quantitative PCR (RT-qPCR) to determine MMP1 expression level in HCC. The procedures were as follows:

Samples inclusion: 108 cases diagnosed HCC pathologically at Ningbo University affiliated Lihuili hospital, Ningbo, from 2012 to 2020, were included into this study. We obtained paired samples of tumor and adjacent tissues (normal) from HCC patients by surgical resection and stored in liquid nitrogen.

WB: We randomly selected 20 of the samples. Cells were lysed in ice-cold radioimmunoprecipitation assay (RIPA) cell lysis buffer supplemented with phenylmethanesulfonyl fluoride (PMSF) (Beyotime Biotechnology, Shanghai, China). Protein was extracted and quantitated by BCA protein assay kit (Beyotime Biotechnology, Shanghai, China). Equal proteins were separated by 10% sodium dodecyl sulfate polyacrylamide gel electrophoresis (SDS-PAGE). Then the targeted blots were cut and electro-transferred to polyvinylidene difluoride (PVDF) membranes (Millipore, MA, USA). Membranes were blocked with 5% fat-free milk in Tris-buffered saline containing 0.05% Tween-20 at room temperature for 1 h, followed by incubation with anti-MMP1 (1:5000; cat. no. ab38929; Abcam) and anti-GAPDH (1:10,000; cat. no. ab8245; Abcam) overnight at 4 °C. Corresponding secondary antibodies were co-incubated at 4 °C for 1 h, followed by dilution in PBS. The protein bands were semi-quantified and photographed by the AlphaView analysis system (ProteinSimple, CA, USA).

RT-qPCR: Total RNA was extracted from samples using TRIzol^®^ LS (Invitrogen, CA, USA). cDNA was synthesized with PrimeScript™ RT reagent kit with gDNA Eraser (cat#RR047A; TAKARA-bio) and amplified with SYBR^®^ Premix Ex Taq™ II kit (cat#RR820A; TAKARA-bio) on the ABI PRISM^®^ 7500 Sequence Detection System (Applied Biosystems, CA, USA), according to the manufacturers’ protocols. Sequences of primers (all purchased from Sangon Biotech (Shanghai) Co., Ltd.) were as follows: MMP1 (RefSeq: NM_002421.4): Fwd 5’- ATGCGAACAAATCCCTTCTACC-3’; Rev 5’- TTTCCTCAGAAAGAGCAGCATCG -3’); β-actin (RefSeq: NM_001101.5): Fwd 5’-CCTTCCTGGGCATGGAGTCCTG-3’; Rev 5’-GGAGCAATGATCTTGATCTTC-3’. The mixture was incubated at 95 °C for 30 s, followed 40 alternate cycles at 95 °C for 5 s and 60 °C for 34 s. The 2^−ΔΔCT^ method^51^ was applied to semiquantitative gene expression analysis with normalized level of β-actin.

### Clinical verification of MMP1-related prognostic model

Clinical data of 108 patients above was retrieved. We evaluated the baseline characteristics and conducted survival analysis to determine OS and PFS. Using the same clinical indicators, we conducted the univariate and multivariate analyses for the OS, as well as the MMP1-related nomogram prognostic model. Corresponding validation of time dependent ROC curve, DCA and prognostic calibration analysis were subsequently performed.

### Correlation analysis of MMP1and tumor-immune microenvironment in LIHC

We conducted the correlation analysis of MMP1 expression with 24 immune-related cells^52^, as well as their infiltration levels in LIHC using the spearman’s test^53^. Similar analysis of other TIICs was visualized via TIMER 2.0 with different algorithms. We also explored the clinical relevance of tumor immune subsets. Relationships between MMP1 and three kinds of immunomodulators^54^ expression across different cancers based on TCGA were visualized using heatmaps. After filtering out the genes with *p* > 0.05, we conducted Lasso regression and prognostic risk factors prediction of immuno-inhibitors, immune-stimulators and major histocompatibility complex (MHC) molecules.

### Evaluation of therapy

We investigated the correlation between MMP1 expression levels and the tumor mutational burden (TMB) and microsatellite instability (MSI) in pan-carcinomas using Sangerbox tools (http://www.sangerbox.com), a free online platform for data analysis. Immunotherapy analysis and drug evaluation were performed accordingly via tumor-immune system interaction database (TISIDB)^55^, integrating multiple heterogeneous data. Immunophenotyping score (IPS) was included to further estimate the correlation between MMP1 and immunotherapy.

### Statistical analysis

All statistical analysis and graphing were performed using R (version 3.6.3). Normally distributed variables were analyzed using the t-test and one-way ANOVA test. Non-normally distributed variables were analyzed using nonparametric tests. Log-rank test and cox regression were used for survival analysis, Pearson’s correlation and spearman’s rank correlation test for correlation analysis. *p* value < 0.05 was considered statistically significant. The correlations was defined as follows: 0.00–0.10 (negligible), 0.10–0.39 (weak), 0.40–0.69 (moderate), 0.70–0.89 (strong), 0.90–1.00 (very strong)^56^.

### Ethical approval

All procedures in the study involving human participants were approved by the research ethics committee of Lihuili hospital affiliated to Ningbo University with informed consent written from each patient prior to enrollment (Approval no. KY2021PJ082). This study was performed in compliance with the 1964 Helsinki declaration and its later amendments or comparable ethical standards.

## Results

### Overview of MMP1

**Figure 1:**
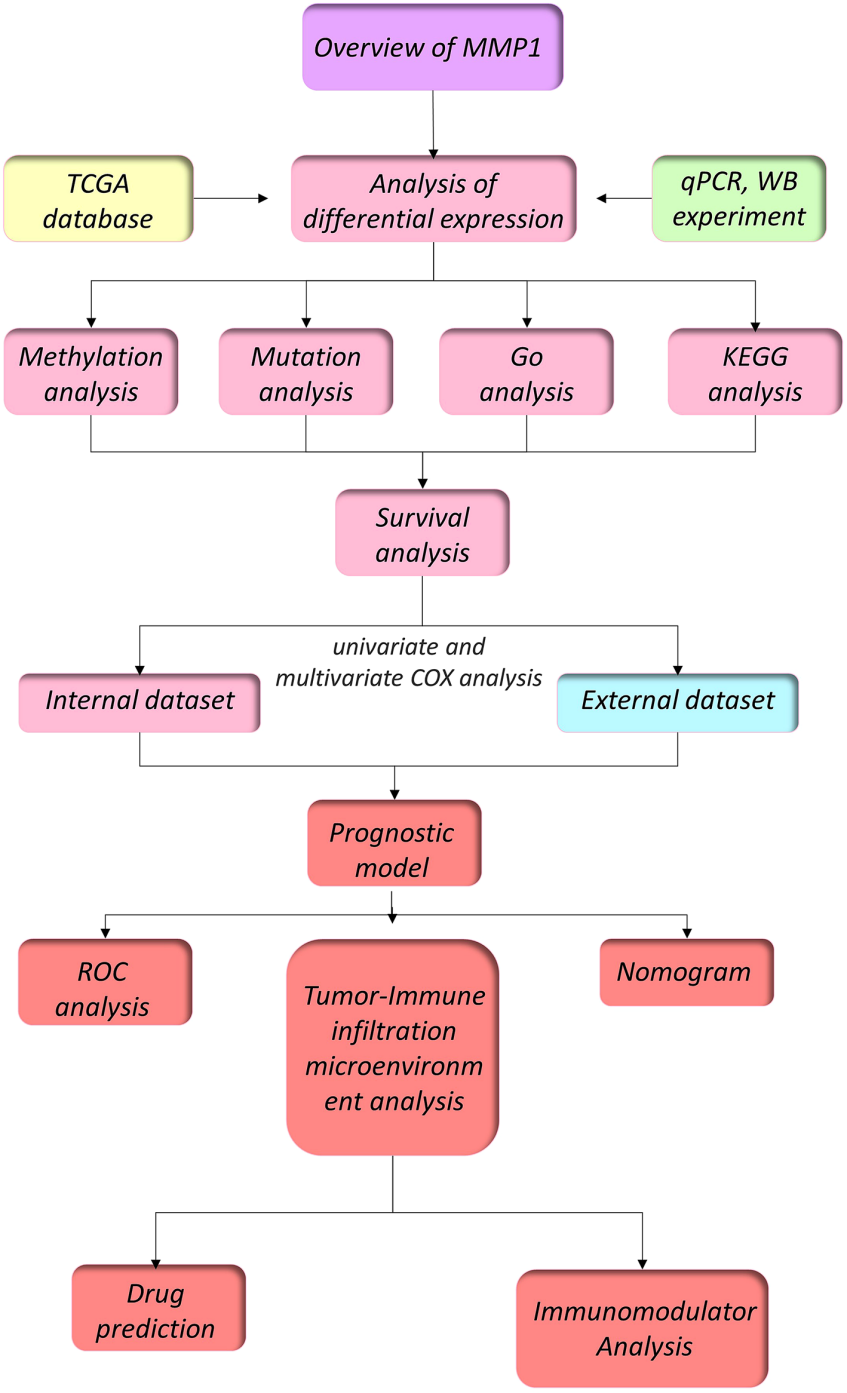
Study design flow chart. TCGA, the cancer genome atlas; qPCR, quantitative Polymerase Chain Reaction; WB, western blot; ROC, receiver operating characteristic curve.

**Figure 2:**
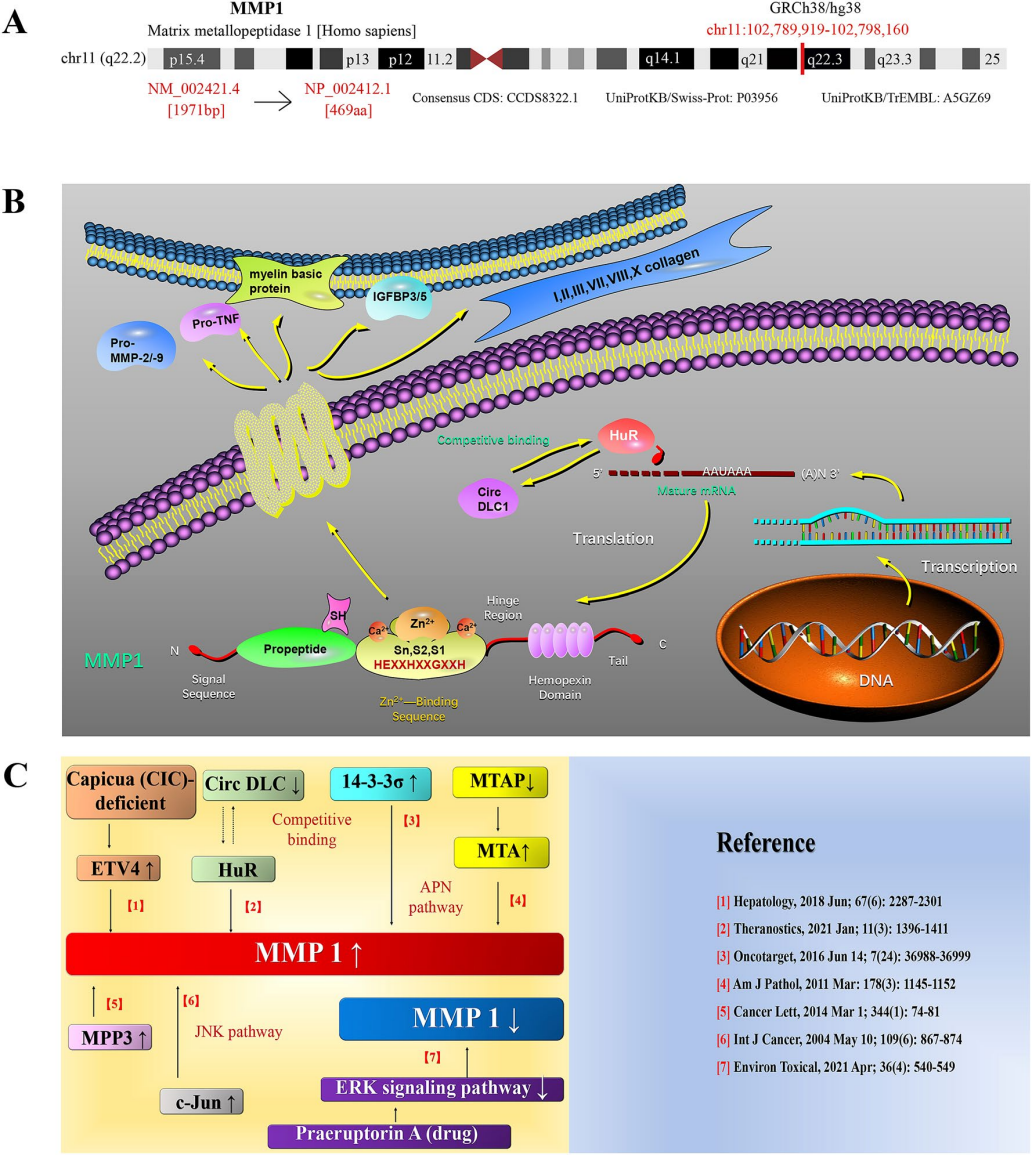
MMP1 gene information, mechanisms and pathways. (**A**) Genomic location and structural characteristics of human MMP1; (**B**) Potential mechanisms and pathways of MMP1; (**C**) The reported pathogenic pathways mediated by MMP1 in multiple cancers with relevant references included.

The study design is detailed in the flow chart in Fig. 1. The study aimed to perform a multidimensional assessment of the tumorigenic role and prognostic potential of human MMP1 (NM_002421.4 for mRNA or NP_002412.1 for protein, Fig. 2A). We refined and summarized MMP1 mechanisms and pathways published in recent years (Fig. 2). MMP1 is a zinc-dependent endopeptidase with the function of degrading multiple substrates (e.g., I, II, III, VII collagen, pro-TNF, pro-MMP2, etc.) in the tumor microenvironment^57^ (Fig. 2B). According to the reported studies so far, most inferential mechanisms and pathways based on experiments revealed that up-regulation of MMP1 expression led to tumor progression, involving the down-expression of circ DLC1^58^, capicua (CIC)^59^ and MTAP^60^, and the up-expression of 14–3-3σ^61^, MPP3^62^ and c-Jun^63^. On the flip side, Praeruptorin A was considered to reduce metastasis of HCC via ERK/MMP1 signaling pathway^64^ (Fig. 2C).

### Comparative analysis of MMP1 expression

**Figure 3:**
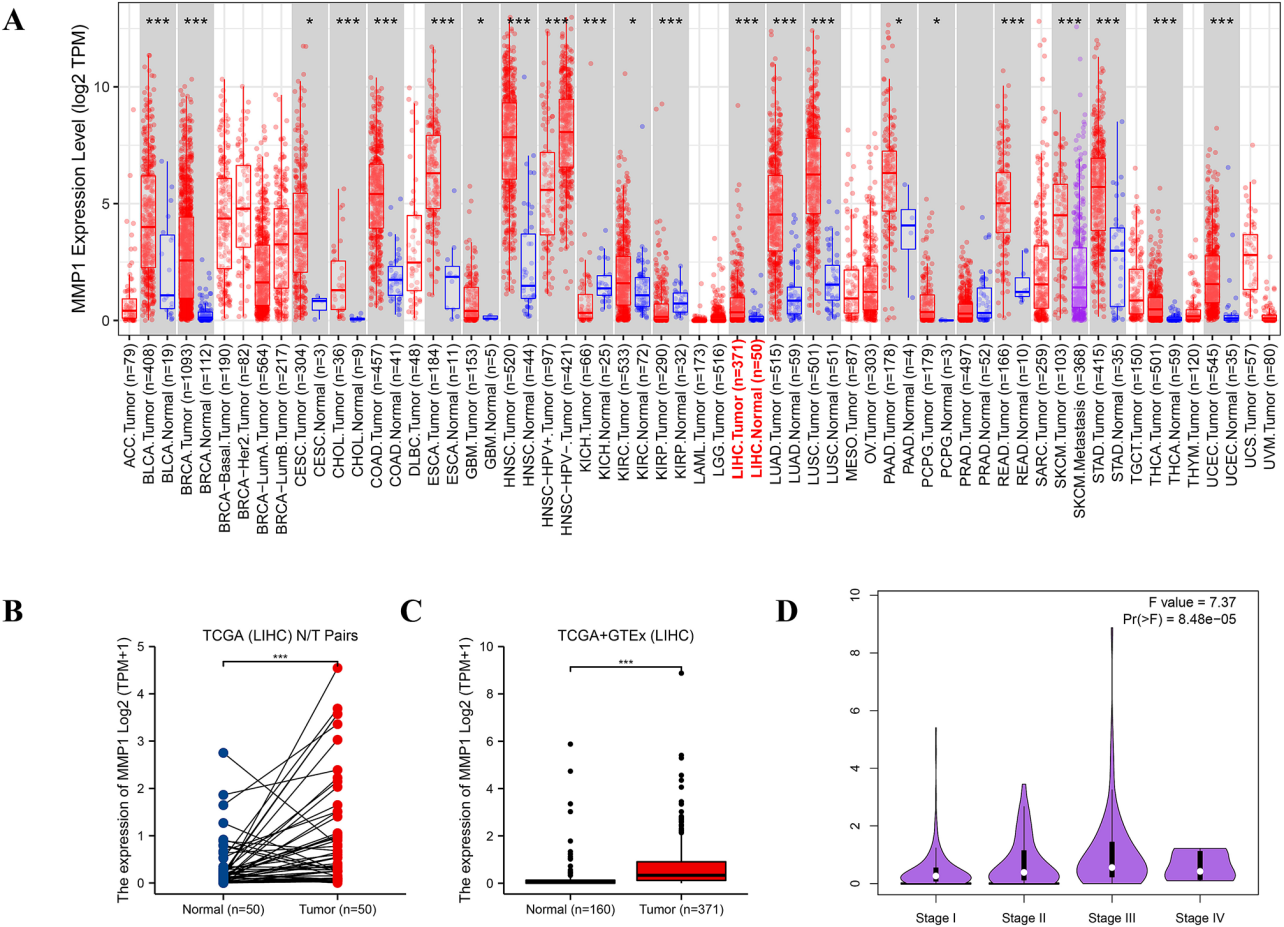
MMP1 expression levels in different tumors, tissues and pathological stages. (**A**) Analysis of MMP1 expression level in different tumors or their subtypes and corresponding normal tissues via TIMER2.0. (**B**) Paired comparison of MMP1 expression level in LIHC. (**C**) Unpaired comparison of MMP1 expression level in LIHC by including the relevant normal tissues of the GTEx database as controls. (**D**) Analysis of MMP1 expression in different pathological stages of LIHC. LIHC, liver hepatocellular carcinoma. *P < 0.05, **P < 0.01, ***P < 0.001.

We compared MMP1 expression levels between tumor and normal tissues in all cancer types of TCGA via TIMER2.0. As shown in Fig. 3A, the MMP1 expression level in the tumor tissues of LIHC was much higher than the adjacent normal tissues (*P* < 0.001). We proceeded to conduct Wilcoxon signed rank test on 50 matched pairs sample in TGCA (Fig. 3B) and included data of the normal tissues from the GTEx database as controls (corrected the batch effects via TOIL method) to further evaluate the difference in MMP1 expression between tumor and normal tissues of LIHC (Fig. 3C). MMP1 expression was consistently elevated in tumor than normal tissues in LIHC (*P* < 0.001). Based on GEO database, it was further verified that MMP1 expression was higher in tumor tissues than normal tissues in GSE14520 (*P* < 0.001) (Fig. S1A) and GSE 25,097 (*P* < 0.05) (Fig. S1B). We also investigated the differential expression level of MMP1 in each pathological stage of LIHC using “pathological stage plot” module of GEPIA2.0. Significant differences were observed (F value = 7.37, *P* = 8.48e-05) (Fig. 3D).

### Genetic alteration analysis

**Figure 4:**
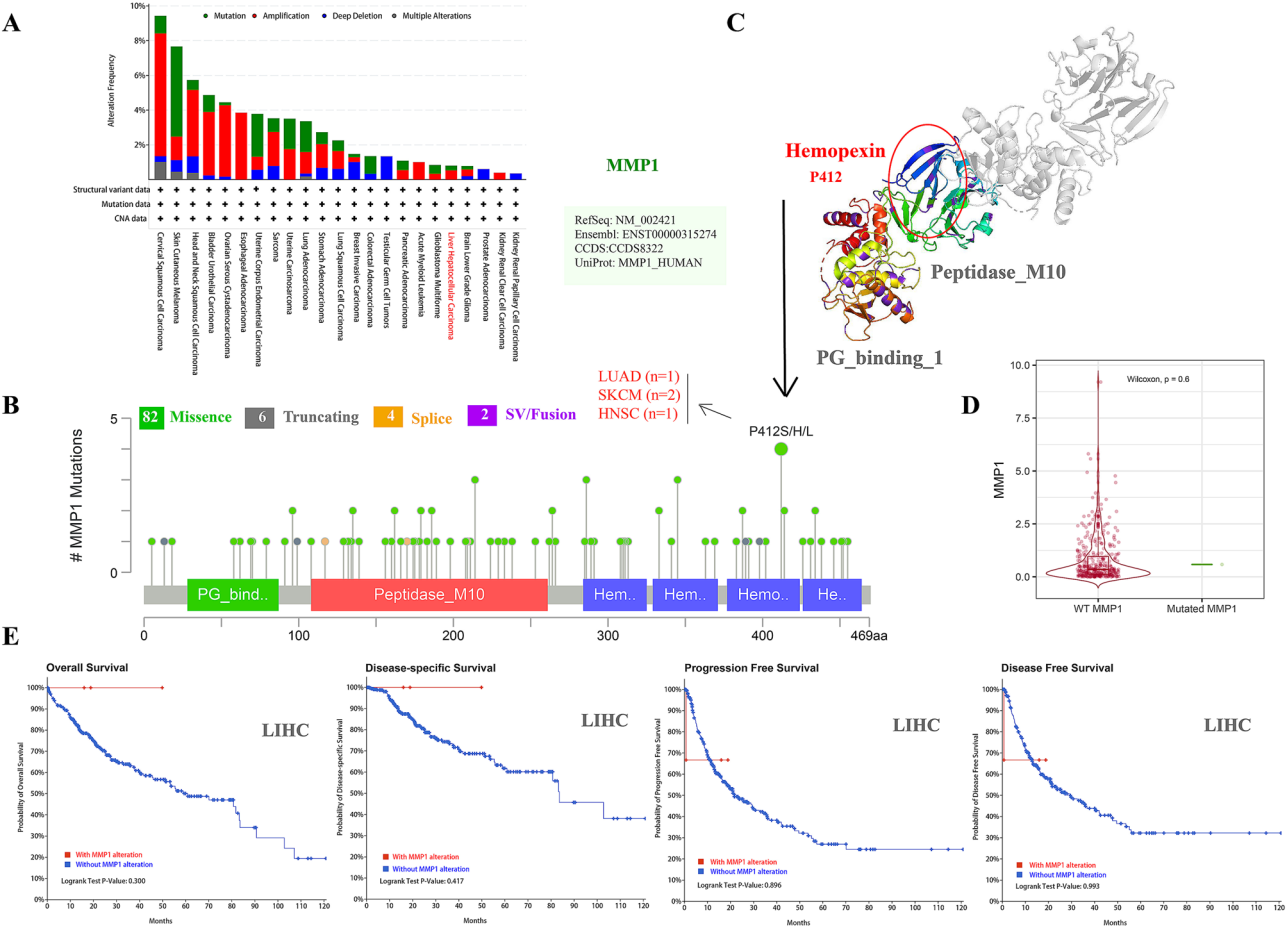
Genetic variation features of MMP1 in different cancers based on TCGA via cBioPortal. (**A**) The mutation frequency and mutation types in diverse cancers; (**B**) Potential sites of mutation; (**C**) mutation site with highest frequency (P412S/H/L) in the 3D version of MMP1 and related carcinomas; (**D**) Comparison of expression level between mutated and WT MMP1; (E) MMP1 alteration impact on OS, DSS, DFS, and PFS of liver hepatocellular carcinoma (LIHC) by the cBioPortal. WT, wild type; OS, overall survival; DSS, disease-specific survival; DFS, disease free survival; PFS, progression free survival; *P < 0.05.

Using the cBioPortal, we conducted a comprehensive analysis of genetic alteration status of MMP1 across different cancers in the TCGA database. Although the alteration frequency of MMP1 in LIHC was lower than 2%, amplification was the dominant component (Fig. 4A). We also observed that mutation was the only other type of MMP1 genovariation in LIHC. Detailed information of mutation sites, types and frequencies of MMP1 are shown in Fig. 4B. Missense mutation of MMP1 was the most common form (82/94, 87.23%) and P412S/H/L mutation in the Hemopexin domain was detected in 2 cases of skin cutaneous carcinoma (SKCM), 1 case of lung adenocarcinoma (LUAD) and 1 case of head and neck squamous cell carcinoma (HNSC) (Fig. 4B), which may result in frame-shift mutation of the MMP1, translation from S (serine) to H (histidine)/L (leucine) at the 412 site of MMP1 protein and promoting protein truncation. The 3D structure MMP1 with the mutated portion is presented in Fig. 4C. Subsequently, we assessed the expression difference between wild type (WT) and mutated MMP1 (*P* = 0.6, Fig. 4D), as well as the relationship between MMP1 alteration and prognosis in LIHC. As demonstrated in Fig. 4E, there was no statistical significance between LIHC patients with and without MMP1 alteration on OS, DSS, PFS and DFS (all *P* > 0.05).

### DNA methylation and gene enrichment analysis

**Figure 5:**
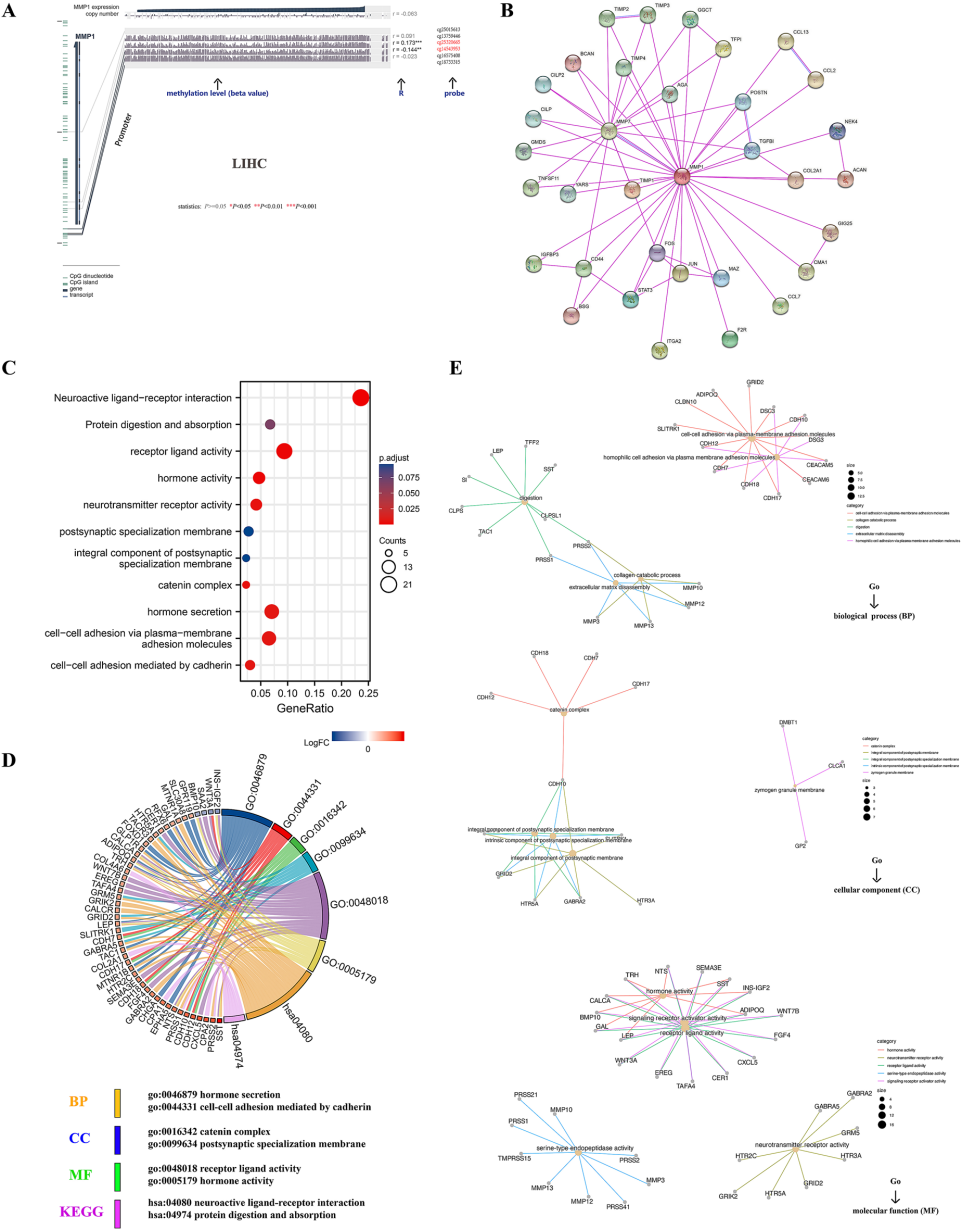
MMP1 DNA methylation in TCGA-LIHC and Gene enrichment analysis. (**A**) Correlation between MMP1 DNA methylation and LIHC expressed as beta value, Pearson correlation coefficients (R) and Benjamini-Hochberg-adjusted *P* value; (**B**) MMP1-related functional proteins association networks with experimental determination via STRING database; (**C**) MMP1-related genes KEGG pathway analysis; (**D**) MMP1-related genes Go analysis; (**E**) Centplots for molecular function (MF), cellular component (CC) and biological process (BP) data.

With the aid of MEXPRESS, we investigated the potential correlation between MMP1 DNA methylation and tumorigenesis in LIHC. Despite the inadequate methylation data, we were still able to observe some significant differences in several probes. A significant negative correlation at probe (cg14543953) of promoter region (r =  0.144, *P* < 0.01) and positive correlation at probe (cg25320665) of non-promoter region (r = 0.173, *P* < 0.001) were observed (Fig. 5A).

For further exploration of MMP1 molecular mechanism in tumorigenicity, we tried to construct MMP1-related PPI networks and conduct enrichment analyses of the signal pathways. 50 MMP1-binding proteins verified by experimental evidence were acquired and their interaction network chart via STRING tool (Fig. 5B). By screening out genes with low correlations, we obtained 300 highly correlated genes (log2FC > 2/ <  2 and p value < 0.05) to conduct KEGG and GO enrichment analysis. We acquired 1 dataset of KEGG, 9 datasets of MF, 1 dataset of CC and 18 datasets of BP under the threshold conditions (adjust *P* < 0.05 and q value < 0.2). KEGG plot suggested that “neuroactive ligand-receptor interaction” (*P* < 0.001) might be the main pathways involved in MMP1 tumorigenicity, as well as “catenin complex” (*P* = 0.004) of CC. Based on the GO enrichment analysis, “hormone secretion”, “cell–cell adhesion mediated by cadherin” and “cell–cell adhesion via plasma-membrane” (all *P* = 0.006) of BP and “receptor ligand activity” (*P* = 0.001), “hormone activity” (*P* = 0.001) and “neurotransmitter receptor activity” (*P* = 0.005) of MF were predicted to have intimate connection to MMP1 (Fig. 5C). We included the log2FC for conjoint analysis to perform a chordal graph showing highly related genes in KEGG/GO datasets. We observed that most of them had a significant positive correlation with MMP1, while the other four had a negative correlation, including INS-IGF2, WNT3A, SAA2 and BMP10 (Fig. 5D). Upon drilling down the GO analysis, several potential signal pathways were also predicted, such as digestion, collagen catabolic process and extracellular matrix disassembly of BP, zymogen granule membrane of CC and serine-type endopeptidase activity of MF (Fig. 5E).

### Clinical correlation and Survival analysis

**Figure 6:**
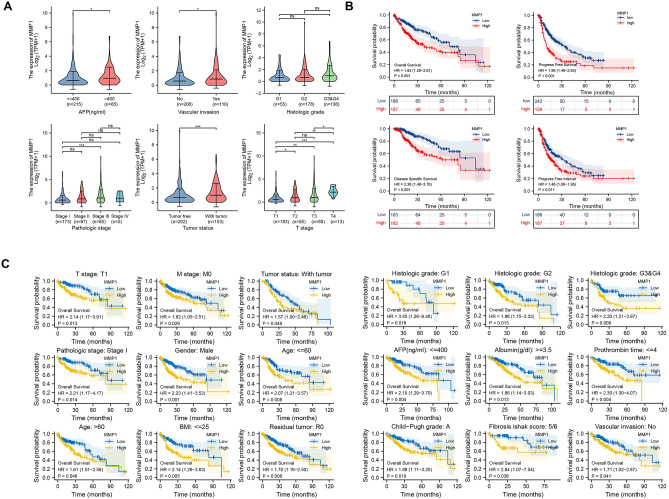
Clinical correlation and prognosis analysis of MMP1 expression in TCGA-LIHC. (**A**) Correlation analysis between multiple clinical indicators and MMP1 expression; (**B**) Prognosis analysis of MMP1 expression in TCGA-LIHC; (**C**) A series of subgroup analyses on OS of MMP1 expression in TCGA-LIHC. OS, overall survival, *P < 0.05, **P < 0.01, ***P < 0.001.

374 cases of LIHC from TCGA were divided equally into two groups according to expression level of MMP1 (low (0–50%) vs high (50–100%)). The patients’ background and baseline characteristics are summarized in Table 1. There were significant differences between the groups with respect to T stage (*P* < 0.001), pathologic stage (*P* = 0.012), tumor status (*P* = 0.013), histologic grade (*P* = 0.01) and vascular invasion (*P* = 0.007). There was no significant difference in other clinical characteristics (Table1). We evaluated the correlation between MMP1 expression and clinical indicators one by one and identified 6 associated risk factors. MMP1 expression was higher in patients with AFP > 400 ng/ml than those with AFP ≤ 400, as well as in patients with vascular invasion than ones without (all *P* values < 0.05). As for histologic grade, higher expression of MMP1 was only observed in G3&G4 compared to G1 (*P* < 0.05). There was significant difference between pathologic stage III and stage I (*P* < 0.001). Patients with tumor had a higher MMP1 expression level than tumor free patients (*P* < 0.001). T stage might be a highly sensitive clinical indicator correlated with MMP1 since there was significant difference in the expression level between each stage (T1 vs T2, *P* < 0.05; T1 vs T3, *P* < 0.01; T1 vs T4, *P* < 0.001; T2 vs T4, *P* < 0.01; T3 vs T4, *P* < 0.05) except T2 vs T3 (Fig. 6A). Furthermore, we included these 6 indicators logistic regression analysis. T stage (Odds Ratio (OR) = 1.985 (1.316–3.009), *P* = 0.001), vascular invasion (OR = 1.952 (1.225–3.127), *P* = 0.005), histologic grade (OR = 2.072 (1.151–3.833), *P* = 0.017), tumor status (OR = 1.754 (1.149–2.688), *P* = 0.009) and pathologic stage (OR = 1.862 (1.221–2.853), *P* = 0.004) were determined to be risk factors associated with MMP1 in LIHC patients (Table 2).

**Table 1:** Baseline characteristics of patients (TCGA-LIHC). Data are presented as n (%).

Characteristics	Levels	Low expression of MMP1	High expression of MMP1	*p* value*
		N = 187	N = 187	
Age (no. (%))^#^	≤ 60	87 (46.8%)	90 (48.1%)	0.874
	> 60	99 (53.2%)	97 (51.9%)	
Gender (no. (%))	Female	60 (32.1%)	61 (32.6%)	1.000
	Male	127 (67.9%)	126 (67.4%)	
T stage (no. (%))	T1	107 (57.8%)	76 (40.9%)	**< 0.001**
	T2	44 (23.8%)	51 (27.4%)	
	T3	33 (17.8%)	47 (25.3%)	
	T4	1 (0.5%)	12 (6.5%)	
N stage (no. (%))	N0	123 (98.4%)	131 (98.5%)	1.000
	N1	2 (1.6%)	2 (1.5%)	
M stage (no. (%))	M0	133 (99.3%)	135 (97.8%)	0.622
	M1	1 (0.7%)	3 (2.2%)	
Pathologic stage (no. (%))	Stage I	99 (57.2%)	74 (41.8%)	**0.012**
	Stage II	41 (23.7%)	46 (26%)	
	Stage III	31 (17.9%)	54 (30.5%)	
	Stage IV	2 (1.2%)	3 (1.7%)	
Tumor status (no. (%))	Tumor free	114 (63.7%)	88 (50%)	**0.013**
	With tumor	65 (36.3%)	88 (50%)	
BMI (no. (%))	≤ 25	90 (52%)	87 (53%)	0.937
	> 25	83 (48%)	77 (47%)	
Residual tumor (no. (%))	R0	170 (96%)	157 (93.5%)	0.217
	R1	6 (3.4%)	11 (6.5%)	
	R2	1 (0.6%)	0 (0%)	
Histologic grade (no. (%))^^^	G1	36 (19.4%)	19 (10.4%)	**0.010**
	G2	95 (51.1%)	83 (45.4%)	
	G3 + G4	55 (29.5%)	81 (44.2%)	
AFP(ng/ml) (no. (%))	≤ 400	119 (80.4%)	96 (72.7%)	0.168
	> 400	29 (19.6%)	36 (27.3%)	
Albumin(g/dl) (no. (%))	< 3.5	34 (21.2%)	35 (25%)	0.527
	≥ 3.5	126 (78.8%)	105 (75%)	
Prothrombin time (no. (%))	≤ 4	113 (72.4%)	95 (67.4%)	0.410
	> 4	43 (27.6%)	46 (32.6%)	
Child–Pugh grade (no. (%))	A	123 (91.8%)	96 (89.7%)	0.640
	B	11 (8.2%)	10 (9.3%)	
	C	0 (0%)	1 (0.9%)	
Fibrosis ishak score (no. (%))	0	41 (34.5%)	34 (35.4%)	0.528
	1/2	14 (11.8%)	17 (17.7%)	
	3/4	15 (12.6%)	13 (13.5%)	
	5/6	49 (41.2%)	32 (33.3%)	
Vascular invasion (no. (%))	No	127 (72.2%)	81 (57%)	**0.007**
	Yes	49 (27.8%)	61 (43%)	

*Compared with each group (Fisher exact test, or Pearson’s chi-square test). #Compared with Wilcoxon rank sum test. ^Compared with Kruskal–wallis Test. *p* value < 0.05 was considered statistically significant (highlighted in bold).

**Table 2:** Logistics regression model of MMP1 (TCGA-LIHC).

Characteristics	Total(N)	Odds Ratio (OR)	*P* value*
T stage (T2&T3&T4 vs. T1)	371 (188 vs. 183)	1.985 (1.316–3.009)	**0.001**
Vascular invasion (Yes vs. No)	318 (110 vs. 208)	1.952 (1.225–3.127)	**0.005**
AFP (ng/ml) (> 400 vs. ≤ 400)	280 (65 vs. 215)	1.539 (0.882–2.703)	0.130
Histologic grade (G2&G3&G4 vs. G1)	369 (314 vs. 55)	2.072 (1.151–3.833)	**0.017**
Tumor status (With tumor vs. Tumor free)	355 (153 vs. 202)	1.754 (1.149–2.688)	**0.009**
Pathologic stage (Stage II&III&IV vs. Stage I)	350 (177 vs. 173)	1.862 (1.221–2.854)	**0.004**

*p* value < 0.05 was considered statistically significant (highlighted in bold).

We had access to data from TCGA and Kaplan–Meier plotter to investigate the prognostic potential of MMP1 expression in LIHC. We found that higher expression of MMP1 was associated with a poorer prognosis in patients with LIHC (OS: HR = 1.83, 95%CI = 1.29–2.61, *P* = 0.001, n = 373; PFS: HR = 1.96, 95%CI = 1.46–2.63, *P* < 0.001, n = 370; DSS: HR = 2.36, 95%CI = 1.48–3.76, *P* < 0.001, n = 365; PFI: HR = 1.46, 95%CI = 1.09–1.95, *P* = 0.011, n = 373. Figure 6B). We subsequently conducted subgroup survival analysis for an in-depth evaluation of the correlation between MMP1 expression and various of clinicopathological factors (Table 3). Elevated MMP1 expression was associated with a worse OS in T1 stage (HR = 2.14, 95%CI = 1.17–3.19, *P* = 0.013), M0 stage (HR = 1.62, 95%CI = 1.05–2.51, *P* = 0.029), tumor-bearing (HR = 1.57, 95%CI = 1.00–2.46, *P* = 0.049), pathologic stage I (HR = 2.21, 95%CI = 1.17–4.17, *P* = 0.014), males (HR = 2.23, 95%CI = 1.41–3.52, *P* = 0.001), age ≤ 60 (HR = 2.07, 95%CI = 1.21–3.57, *P* = 0.008) or age > 60 (HR = 1.61, 95%CI = 1.01–2.56, *P* = 0.046), BMI ≤ 25 (HR = 2.14, 95%CI = 1.26–3.63, *P* = 0.005), R0 resection (HR = 1.70, 95%CI = 1.16–2.50, *P* = 0.006), histologic G1 (HR = 3.45, 95%CI = 1.26–9.46, *P* = 0.016)/G2 (HR = 1.96, 95%CI = 1.15–3.32, *P* = 0.013)/G3&G4 (HR = 2.20, 95%CI = 1.21–3.97, *P* = 0.009), AFP ≤ 400 (HR = 2.19, 95%CI = 1.29–3.70, *P* = 0.004), Albumin ≥ 3.5 (HR = 1.86, 95%CI = 1.14–3.03, *P* = 0.013), Prothrombin time ≤ 4 (HR = 2.30, 95%CI = 1.30–4.07, *P* = 0.004), Child–Pugh A (HR = 1.88, 95%CI = 1.11–3.20, *P* = 0.019), fibrosis ishak of 5/6 (HR = 2.84, 95%CI = 1.07–7.54, *P* = 0.036) and nonvascular invasion (HR = 1.71, 95%CI = 1.02–2.87, *P* = 0.041) (Fig. 6C).

**Table 3:** Correlation of MMP1 expression and OS in hepatocellular carcinoma patients with different clinicopathological factors via Kaplan–Meier plotter.

Clinicopathological factors	Subgroup	Overall survival		
		No	Hazard ratio (95% CI)	*P* value*
T stage	T1	183	2.14 (1.17–3.91)	**0.013**
	T2	95	1.28 (0.62–2.65)	0.499
	T3	80	1.77 (0.96–3.26)	0.065
	T4	13	0.37 (0.09–1.55)	0.173
N stage	N0	254	1.51 (0.98–2.33)	0.064
	N1	4	NA	NA
M stage	M0	268	1.62 (1.05–2.51)	**0.029**
	M1	4	NA	NA
Pathologic stage	Stage I	173	2.21 (1.17–4.17)	**0.014**
	Stage II	87	1.59 (0.72–3.51)	0.251
	Stage III	85	1.78 (0.98–3.22)	0.059
	Stage IV	5	NA	NA
Tumor status	Tumor free	202	1.76 (0.95–3.28)	0.074
	With tumor	153	1.57(1.00–2.46)	**0.049**
Gender	Male	253	2.23 (1.41–3.52)	**0.001**
	Female	121	1.31 (0.75–2.28)	0.348
Age	≤ 60	177	2.07 (1.21–3.57)	**0.008**
	> 60	196	1.61 (1.01–2.56)	**0.046**
BMI	≤ 25	177	2.14 (1.26–3.63)	**0.005**
	> 25	160	1.35 (0.78–2.36)	0.284
Residual tumor	R0	327	1.70 (1.16–2.50)	**0.006**
	R1	17	1.00 (0.21–4.71)	0.996
	R2	1	NA	NA
Histologic grade	G1	55	3.45 (1.26–9.46)	**0.016**
	G2	178	1.96 (1.15–3.32)	**0.013**
	G3&G4	136	2.20 (1.21–3.97)	**0.009**
AFP(ng/ml)	≤ 400	215	2.19 (1.29–3.70)	**0.004**
	> 400	65	2.14 (0.89–5.12)	0.088
Albumin(g/dl)	< 3 5	69	1.51 (0.64–3.53)	0.347
	≥ 3 5	231	1.86 (1.14–3.03)	**0.013**
Prothrombin time	≤ 4	208	2.30 (1.30–4.07)	**0.004**
	> 4	89	0.94 (0.51–1.76)	0.852
Child–Pugh grade	A	219	1.88 (1.11–3.20)	**0.019**
	B	21	1.83 (0.44–7.58)	0.406
	C	1	NA	NA
Fibrosis ishak score	0	75	1.98 (0.92–4.24)	0.079
	1/2	31	0.79 (0.21–2.98)	0.731
	3/4	28	1.70 (0.30–9.61)	0.551
	5/6	81	2.84 (1.07–7.54)	**0.036**
Vascular invasion	No	208	1.71 (1.02–2.87)	**0.041**
	Yes	110	1.20 (0.62–2.33)	0.594

OS overall survival, CI confidence interval, NA not available data. *Compared with each subgroup based on MMP1 expression of 0–50% vs 50–100%. *p* < 0.05 means statistically significant (highlighted in bold).

### Establishment and evaluation of an MMP1 based prognosis prediction model

**Figure 7:**
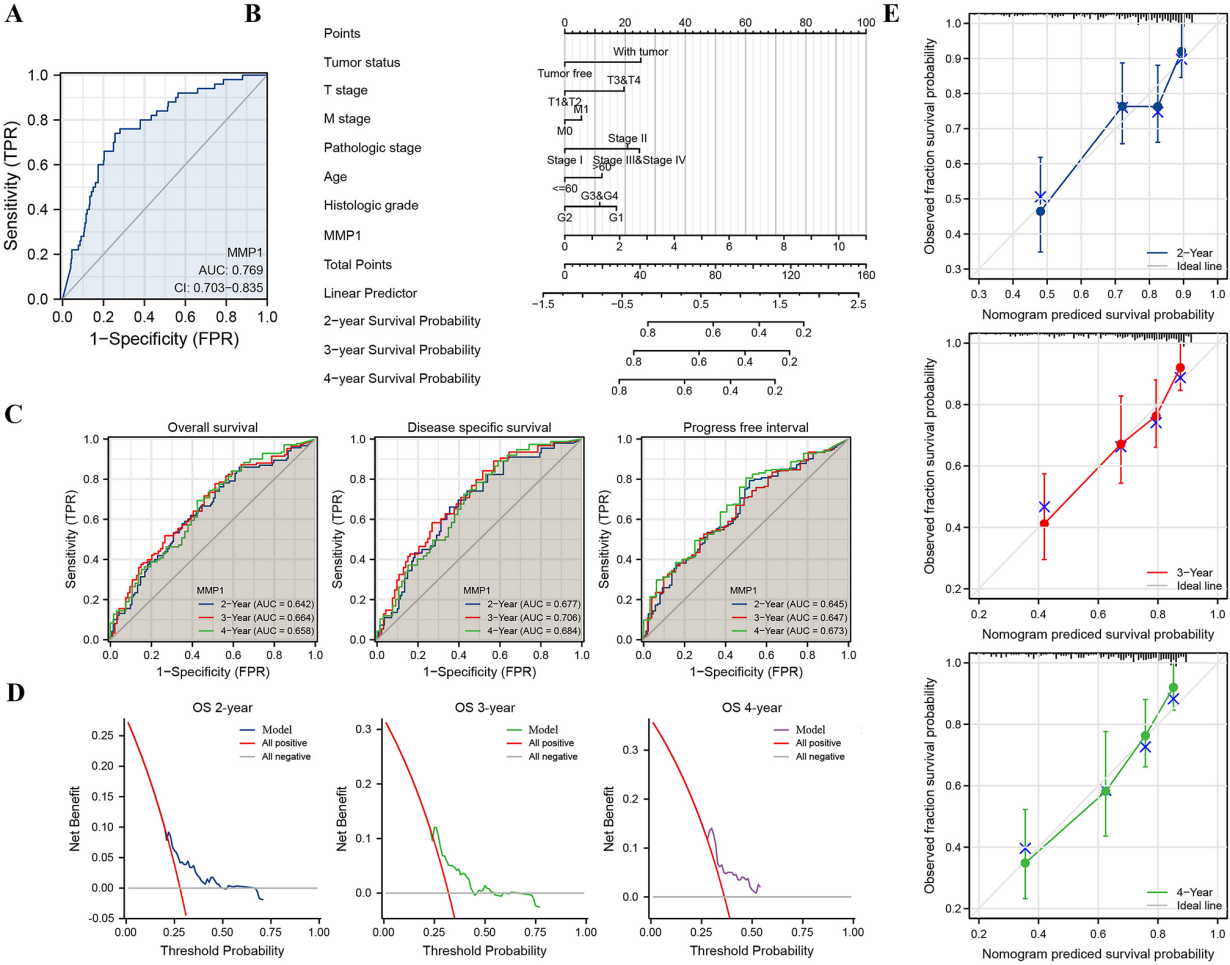
Establishment of an MMP1 expression based prognostic model in TCGA-LIHC. (**A**) Diagnostic ROC curve of MMP1 expression in TCGA-LIHC; (**B**) An MMP1 expression-based nomogram predicting risk of TCGA-LIHC; (**C**) Time dependent ROC curve for verifying the utility of nomogram; (**D**) Decision curve analysis for evaluating the prognostic model; (**E**) Nomogram calibration curve.

According to the TCGA data, we first investigated the diagnostic potential of MMP1 in LIHC and obtained the ROC curve that showed an above average performance (area under the curve (AUC) = 0.769, CI = 0.703–0.835) (Fig. 7A). Next, we included all variables into the univariate analysis in respect to OS, DSS and PFI. T3&T4 stage (*P* < 0.001), M1 stage (*P* = 0.017), pathologic stage III&IV (*P* < 0.001), tumor-bearing status (*P* < 0.001), MMP1 expression (*P* < 0.001) were significantly correlated with OS (Table 4), T3&T4 stage (*P* < 0.001), M1 stage (*P* = 0.024), pathologic stage III&IV (*P* < 0.001), prothrombin time > 4 (*P* = 0.031), Child–Pugh B (*P* = 0.047) and MMP1 expression (*P* < 0.001) to DSS (Table 5) and T3&T4 stage (*P* < 0.001), M1 stage (*P* = 0.035), pathologic stage III&IV (*P* < 0.001), tumor-bearing status (*P* < 0.001), vascular invasion (*P* = 0.003) and MMP1 expression (*P* < 0.001) to PFI (Table 6). Tumor-bearing status (HR = 1.819, 95%CI = 1.137–2.911, *P* = 0.013) and MMP1 expression (HR = 1.236, 95%CI = 1.037–1.473, *P* = 0.018) were independent factors impacting the OS of patients with LIHC (Table 4). Child–Pugh B (HR = 3.667, 95%CI = 1.135–11.845, *P* = 0.030) and MMP1 expression (HR = 1.424, 95%CI = 1.020–1.987, *P* = 0.038) for DSS (Table 5), so as tumor-bearing status (HR = 14.440, 95%CI = 8.484–24.580, *P* < 0.001), vascular invasion (HR = 1.536, 95%CI = 1.009–2.337, *P* = 0.045) and MMP1 expression (HR = 1.204, 95%CI = 1.003–1.446, *P* = 0.047) for PFI (Table 6).

**Table 4:** The univariate and multivariate analysis for the OS (TCGA-LIHC).

Characteristics		Total(N)	Univariate analysis		Multivariate analysis	
			Hazard ratio (95% CI)	*P* value*	Hazard ratio (95% CI)	*P* value*
T stage	T1&T2	277	Reference		NA	
	T3&T4	93	2.598 (1.826–3.697)	** < 0.001**	1.435 (0.195–10.565)	0.723
N stage	N0	254	Reference			
	N1	4	2.029 (0.497–8.281)	0.324		
M stage	M0	268	Reference		NA	
	M1	4	4.077 (1.281–12.973)	**0.017**	1.101 (0.261–4.651)	0.869
Pathologic stage	Stage I	173	Reference		NA	
	Stage II	86	1.416 (0.868–2.312)	0.164	1.562 (0.850–2.870)	0.151
	Stage III&IV	90	2.823 (1.862–4.281)	** < 0.001**	1.971 (0.265–14.666)	0.508
Tumor status	Tumor free	202	Reference		NA	
	With tumor	152	2.317 (1.590–3.376)	** < 0.001**	1.819 (1.137–2.911)	**0.013**
Gender	Male	252	Reference			
	Female	121	1.261 (0.885–1.796)	0.200		
Age	> 60	196	Reference			
	≤ 60	177	0.830 (0.585–1.176)	0.295		
BMI	> 25	159	Reference			
	≤ 25	177	1.253 (0.864–1.819)	0.235		
Residual tumor	R0	326	Reference			
	R1&2	18	1.604 (0.812–3.169)	0.174		
Histologic grade	G1	55	Reference			
	G2	178	1.162 (0.686–1.968)	0.577		
	G3&G4	135	1.222 (0.710–2.103)	0.469		
AFP(ng/ml)	> 400	64	Reference			
	≤ 400	215	0.930 (0.569–1.521)	0.772		
Albumin(g/dl)	≥ 3 5	230	Reference			
	< 3 5	69	1.115 (0.683–1.821)	0.662		
Prothrombin time	≤ 4	207	Reference			
	> 4	89	1.335 (0.881–2.023)	0.174		
Child–Pugh grade	A	218	Reference			
	B	21	1.595 (0.757–3.361)	0.219		
	C	1	2.138 (0.294–15.544)	0.453		
Fibrosis ishak score	0	75	Reference			
	1/2	31	0.935 (0.437–2.002)	0.864		
	3/4	28	0.698 (0.288–1.695)	0.428		
	5/6	80	0.737 (0.410–1.325)	0.308		
Vascular invasion	Yes	109	Reference			
	No	208	0.744 (0.491–1.127)	0.163		
MMP1		373	1.353 (1.199–1.527)	** < 0.001**	1.236 (1.037–1.473)	**0.018**

OS overall survival, CI confidence interval, NA reference group or could not be evaluated. *Compared with each group (Log-Rank test or Omnibus test for univariate, Cox regression analysis with adjusted hazard for multivariate). *p* < 0.05 means statistically significant (highlighted in bold).

**Table 5:** The univariate and multivariate analysis for the DSS.

Characteristics		Total(N)	Univariate analysis		Multivariate analysis	
			Hazard ratio (95% CI)	*P* value*	Hazard ratio (95% CI)	*P* value*
T stage	T1&T2	272	Reference		NA	NA
	T3&T4	90	3.639 (2.328–5.688)	** < 0.001**	1.075 (0.117–9.839)	0.949
N stage	N0	249	Reference			
	N1	4	3.612 (0.870–14.991)	0.077		
M stage	M0	265	Reference		NA	NA
	M1	3	5.166 (1.246–21.430)	**0.024**	2.550 (0.458–14.183)	0.285
Pathologic stage	Stage I&II	254	Reference		NA	NA
	Stage III&IV	87	3.803 (2.342–6.176)	** < 0.001**	2.022 (0.219–18.673)	0.535
Tumor status	Tumor free	202	Reference			
	With tumor	152	775,790,759.389 (0.000-NA)	0.994		
Gender	Male	247	Reference			
	Female	118	1.230 (0.780–1.937)	0.373		
Age	≤ 60	174	Reference			
	> 60	191	0.846 (0.543–1.317)	0.458		
BMI	≤ 25	175	Reference			
	> 25	154	0.826 (0.512–1.330)	0.431		
Residual tumor	R0	320	Reference			
	R1&2	17	1.678 (0.728–3.870)	0.224		
Histologic grade	G1	55	Reference			
	G2	172	1.177 (0.599–2.314)	0.636		
	G3&G4	133	1.228 (0.613–2.462)	0.562		
AFP(ng/ml)	≤ 400	214	Reference			
	> 400	61	0.867 (0.450–1.668)	0.668		
Albumin(g/dl)	< 3 5	67	Reference			
	≥ 3 5	227	1.148 (0.586–2.250)	0.687		
Prothrombin time	≤ 4	203	Reference		NA	NA
	> 4	87	1.778 (1.054–2.999)	**0.031**	1.447 (0.564–3.716)	0.442
Child–Pugh grade	A	214	Reference		NA	NA
	B	20	2.439 (1.011–5.885)	**0.047**	3.667 (1.135–11.845)	**0.030**
	C	1	3.584 (0.484–26.513)	0.211		
Fibrosis ishak score	0	73	Reference			
	1/2	31	1.523 (0.639–3.630)	0.342		
	3/4	28	0.632 (0.183–2.191)	0.470		
	5/6	78	0.788 (0.363–1.708)	0.545		
Vascular invasion	No	204	Reference			
	Yes	105	1.277 (0.707–2.306)	0.418		
MMP1		365	1.389 (1.188–1.624)	** < 0.001**	1.424 (1.020–1.987)	**0.038**

DSS disease specific survival, CI confidence interval, NA reference group or could not be evaluated. *Compared with each group (Log-Rank test or Omnibus test for univariate, Cox regression analysis with adjusted hazard for multivariate). *p* < 0.05 means statistically significant (highlighted in bold).

**Table 6:** The univariate and multivariate analysis for the PFI.

Characteristics		Total(N)	Univariate analysis		Multivariate analysis	
			Hazard ratio (95% CI)	*P* value*	Hazard ratio (95% CI)	*P* value*
T stage	T1&T2	277	Reference		NA	NA
	T3&T4	93	2.177 (1.590–2.980)	** < 0.001**	0.557 (0.125–2.490)	0.444
N stage	N0	254	Reference			
	N1	4	1.370 (0.338–5.552)	0.659		
M stage	M0	268	Reference		NA	NA
	M1	4	3.476 (1.091–11.076)	**0.035**	1.342 (0.381–4.730)	0.647
Pathologic stage	Stage I&II	259	Reference		NA	NA
	Stage III&IV	90	2.201 (1.591–3.046)	** < 0.001**	2.643 (0.590–11.843)	0.204
Tumor status	Tumor free	202	Reference		NA	NA
	With tumor	152	11.342 (7.567–17.000)	** < 0.001**	14.440 (8.484–24.580)	** < 0.001**
Gender	Male	252	Reference			
	Female	121	1.018 (0.747–1.387)	0.909		
Age	≤ 60	177	Reference			
	> 60	196	0.960 (0.718–1.284)	0.783		
BMI	≤ 25	177	Reference			
	> 25	159	0.936 (0.689–1.272)	0.673		
Residual tumor	R0	326	Reference			
	R1&2	18	1.513 (0.840–2.726)	0.168		
Histologic grade	G1	55	Reference			
	G2	178	1.167 (0.750–1.815)	0.494		
	G3&G4	135	1.293 (0.824–2.031)	0.264		
AFP(ng/ml)	≤ 400	215	Reference			
	> 400	64	1.045 (0.698–1.563)	0.832		
Albumin(g/dl)	< 3 5	69	Reference			
	≥ 3 5	230	0.911 (0.618–1.341)	0.636		
Prothrombin time	≤ 4	207	Reference			
	> 4	89	1.100 (0.785–1.541)	0.581		
Child–Pugh grade	A	218	Reference			
	B	21	1.423 (0.761–2.661)	0.270		
	C	1	1.156 (0.161–8.313)	0.885		
Fibrosis ishak score	0	75	Reference			
	1/2	31	1.420 (0.799–2.524)	0.232		
	3/4	28	1.353 (0.746–2.451)	0.319		
	5/6	80	1.345 (0.861–2.101)	0.193		
Vascular invasion	No	208	Reference		NA	NA
	Yes	109	1.676 (1.196–2.348)	**0.003**	1.536 (1.009–2.337)	**0.045**
MMP1		373	1.305 (1.169–1.457)	** < 0.001**	1.204 (1.003–1.446)	**0.047**

PFI progress free interval, CI confidence interval, NA reference group or could not be evaluated. *Compared with each group (Log-Rank test or Omnibus test for univariate, Cox regression analysis with adjusted hazard for multivariate). *p* < 0.05 means statistically significant (highlighted in bold).

According to the univariate and multivariate cox regression analysis above, MMP1 were deemed an important independent prognostic factor in addition to clinical factors. These results showed its potential to serve as a reliable and innovative biomarker for patients with LIHC. More importantly, in order to predict the LIHC patients’ prognosis, we constructed a nomogram incorporating MMP1 and multiple clinicopathological characteristics based on the above analysis. As shown in Fig. 7B, we could calculate the score for each variable and combined them to predict the prognosis of patients with LIHC (C-Index = 0.680, 95%CI = 0.647–0.713). As a supplement, we employed time-dependent ROC curves to assess the accuracy of MMP1 for predicting OS, DSS and PFI in LIHC patients. The AUC values for OS (2-year: 0.642, 3-year: 0.664, 4-year: 0.658), DSS (2-year: 0.677, 3-year: 0.706, 4-year: 0.684) and PFI (2-year: 0.645, 3-year: 0.647, 4-year: 0.673) (Fig. 7C). DCA (Fig. 7D) was performed to determine the clinical utility of the nomogram (C-Index = 0.614, 95%CI = 0.577–0.650). Nomogram calibration curves (Fig. 7E) showed a good predictive accuracy of the model.

### Validation of clinical dataset in HCC patients

**Figure 8:**
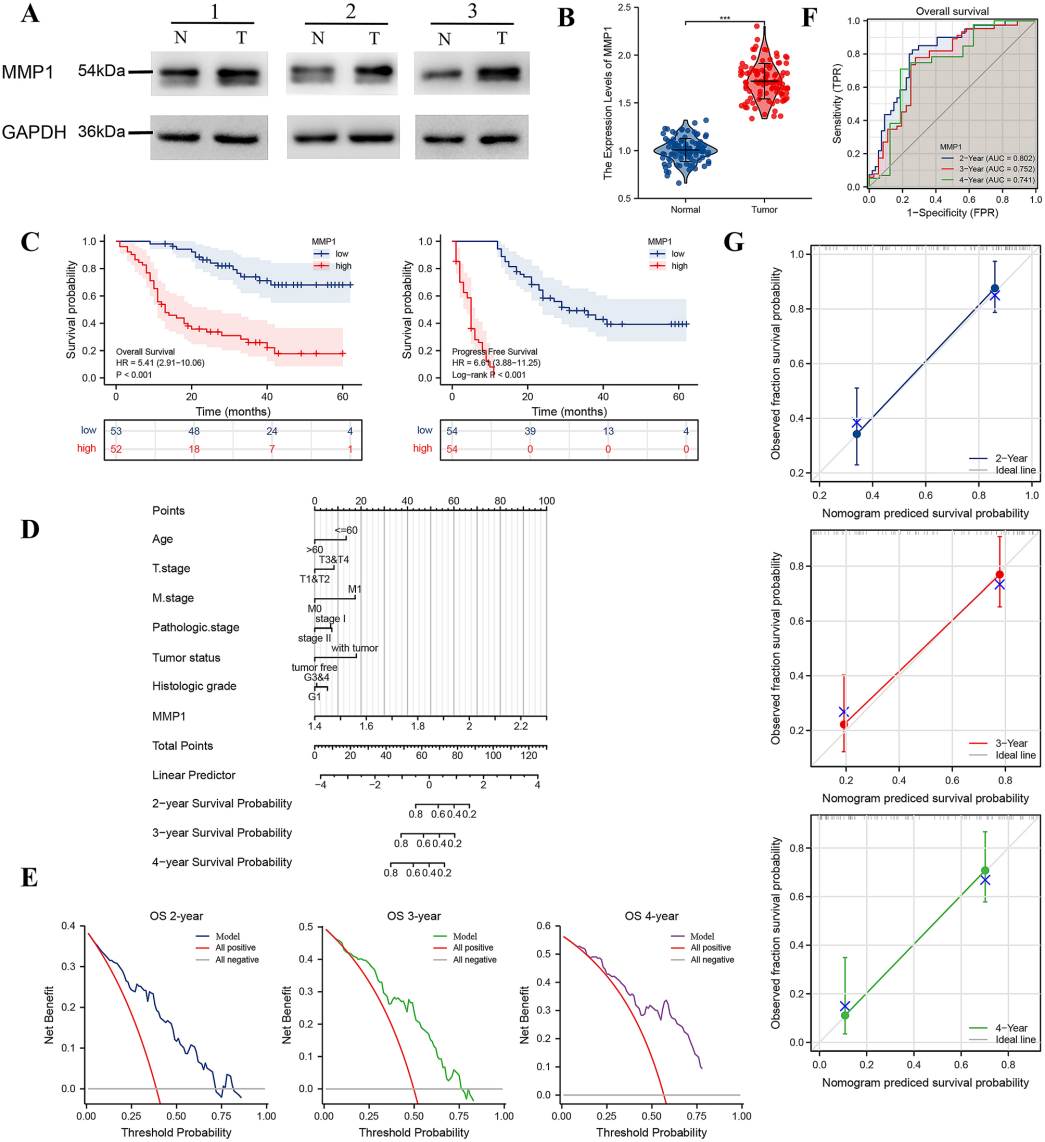
Clinical verification of the MMP1 expression based prognostic model in HCC patients. (**A**) Western blot analysis of MMP1 expression proteins in paired samples from HCC patients (The original images uncropped and the clipping process were shown in supplementary materials); (**B**) qPCR analysis of MMP1 expression in HCC patients; (**C**) Prognosis analysis of MMP1 expression in HCC patients; (**D**) MMP1 expression-based risk prediction nomogram for HCC patients; (**E**) Decision curve analysis of the prognostic model; (**F**) Time dependent ROC curve for accessing the utility of the nomogram; (G) Nomogram calibration curve. HCC, hepatocellular carcinoma. *P < 0.05, **P < 0.01, ***P < 0.001.

Tumor/normal samples and clinicopathological data of 108 patients diagnosed with HCC and treated with surgical resection were retrieved and analyzed. MMP1 expression was higher in tumor tissues at translation level (Fig. 8A). Consistent with the result of WB, higher expression of MMP1 in tumor than normal tissues (*P* < 0.001) were corroborated by qPCR (Fig. 8B).

The 108 cases were divided into two groups according to MMP1 expression level: low (0–50%) and high (50–100%) expression groups. The patients’ background and baseline characteristics are summarized in Table 7. Unlike the baseline of TCGA-LIHC, there were significant differences between the groups with respect to pathologic stage (*P* = 0.019), AFP (*P* = 0.020), albumin (*P* = 0.026) and Child–Pugh grade (*P* = 0.002) in clinical HCC cohort. Consistent with the above results, the Kaplan–Meier survival curves confirmed that the low-expression group was associated with a better OS (HR = 5.41, 95%CI = 2.91–10.06, *P* < 0.001) and PFS (HR = 6.61, 95%CI = 3.88–11.25, *P* < 0.001) (Fig. 8C).

**Table 7:** Baseline characteristics of patients (Clinic HCC). Data are presented as n (%).

Characteristics	Levels	High expression of MMP1	Low expression of MMP1	*p* value*
		N = 54	N = 54	
Age (no. (%))	> 60	19 (35.2%)	25 (46.3%)	0.327
	≤ 60	35 (64.8%)	29 (53.7%)	
Gender (no. (%))	Female	6 (11.1%)	6 (11.1%)	1.000
	Male	48 (88.9%)	48 (88.9%)	
T stage (no. (%))	T1	19 (35.2%)	28 (51.9%)	0.224
	T2	15 (27.8%)	15 (27.8%)	
	T3	13 (24.1%)	8 (14.8%)	
	T4	7 (13%)	3 (5.6%)	
N stage (no. (%))	N0	45 (83.3%)	47 (87%)	0.786
	N1	9 (16.7%)	7 (13%)	
M stage (no. (%))	M0	52 (96.3%)	54 (100%)	0.495
	M1	2 (3.7%)	0 (0%)	
Pathologic stage (no. (%))	Stage I	16 (29.6%)	27 (50%)	**0.019**
	Stage II	8 (14.8%)	13 (24.1%)	
	Stage III	19 (35.2%)	8 (14.8%)	
	Stage IV	11 (20.4%)	6 (11.1%)	
Tumor status (no. (%))	tumor free	36 (66.7%)	42 (77.8%)	0.283
	with tumor	18 (33.3%)	12 (22.2%)	
Residual tumor (no. (%))	R0	41 (75.9%)	47 (87%)	0.376
	R1	4 (7.4%)	2 (3.7%)	
	R2	9 (16.7%)	5 (9.3%)	
Histologic grade (no. (%))	G1	7 (14.6%)	11 (21.2%)	0.145
	G2	16 (33.3%)	24 (46.2%)	
	G3&4	25 (52.1%)	17 (32.7%)	
AFP(ng/ml) (no. (%))	> 400	36 (66.7%)	23 (42.6%)	**0.020**
	≤ 400	18 (33.3%)	31 (57.4%)	
Albumin(g/dl) (no. (%))	< 3.5	19 (35.2%)	8 (14.8%)	**0.026**
	≥ 3.5	35 (64.8%)	46 (85.2%)	
Child–Pugh grade (no. (%))	A	33 (61.1%)	48 (88.9%)	**0.002**
	B	21 (38.9%)	6 (11.1%)	
Vascular invasion (no. (%))	No	28 (53.8%)	35 (66%)	0.282
	Yes	24 (46.2%)	18 (34%)	

*Compared with each group (Fisher exact test, or Pearson’s chi-square test). *p* value < 0.05 was considered statistically significant (highlighted in bold).

Based on univariate logistic regression, Age > 60 (*P* = 0.038), tumor-bearing status (*P* = 0.002), albumin < 3.5 (*P* = 0.004), Child–Pugh B (*P* < 0.001) and MMP1 (*P* < 0.001) were significantly correlated with OS (Table 8). Tumor-bearing status (HR = 3.594, 95%CI = 1.429–9.036, *P* = 0.007) and MMP1 expression (HR = 1.524, 95%CI = 1.238–1.875, *P* < 0.001) were independent factors associated with poor OS, while the age > 60 (HR = 0.501, 95%CI = 0.268–0.939, *P* = 0.031) and R1&2 resection (HR = 0.226, 95%CI = 0.073–0.701, *P* = 0.010) were independent protective factors. Based on the above, we constructed a nomogram composed variables in the MMP1 based nomogram to predict the 2,3and4 years survival probability of HCC patients (Fig. 8D). The model showed a good accuracy for the patients in the cohort (C-Index = 0.797, 95%CI = 0.766–0.828). DCA curves showed consistent results (C-Index = 0.759, 95%CI = 0.727–0.792) (Fig. 8E). The AUC of time-dependent ROC curve likewise showed good performance of the model (0.802, 0.752 and 0.741 at 2, 3 and 4 years, respectively) (Fig. 8F). Nomogram calibration curves are shown in Fig. 8G.

**Table 8:** The univariate and multivariate analysis for the OS (Clinical HCC).

Characteristics		Total(N)	Univariate analysis		Multivariate analysis	
			Hazard ratio (95% CI)	*P* value*	Hazard ratio (95% CI)	*P* value*
Age	≤ 60	62	Reference		NA	
	> 60	43	0.541 (0.303–0.966)	**0.038**	0.501 (0.268–0.939)	**0.031**
Gender	male	93	Reference			
	female	12	1.081 (0.462–2.530)	0.858		
T stage	T1&T2	75	Reference		NA	
	T3&T4	30	1.667 (0.949–2.928)	0.075	1.233 (0.475–3.203)	0.667
N stage	N0	90	Reference			
	N1	15	1.415 (0.690–2.902)	0.343		
M stage	M0	103	Reference		NA	
	M1	2	3.381 (0.815–14.024)	0.093	5.345 (0.901–31.699)	0.065
Pathologic stage	Stage I	42	Reference		NA	
	Stage II	21	0.638 (0.268–1.519)	0.309	0.793 (0.315–1.998)	0.623
	Stage III&IV	42	1.725 (0.958–3.105)	0.069	1.126 (0.421–3.013)	0.813
Tumor status	Tumor free	75	Reference		NA	
	With tumor	30	2.452 (1.395–4.311)	**0.002**	3.594 (1.429–9.036)	**0.007**
Residual tumor	R0	85	Reference		NA	
	R1&2	20	1.774 (0.929–3.389)	0.083	0.226 (0.073–0.701)	**0.010**
Histologic grade	G1	18	Reference			
	G2	39	1.786 (0.720–4.430)	0.211		
	G3&G4	40	2.024 (0.819–5.003)	0.127		
AFP(ng/ml)	> 400	57	Reference			
	≤ 400	48	0.636 (0.366–1.105)	0.108		
Albumin(g/dl)	≥ 3 5	78	Reference		NA	
	< 3 5	27	2.321 (1.317–4.092)	**0.004**	1.242 (0.564–2.738)	0.590
Child–Pugh grade	A	79	Reference		NA	
	B	26	3.069 (1.734–5.431)	** < 0.001**	2.149 (0.942–4.901)	0.069
Vascular invasion	Yes	41	Reference		NA	
	No	61	0.617 (0.355–1.072)	0.087	0.785 (0.418–1.475)	0.452
MMP1		105	1.529 (1.290–1.813)	** < 0.001**	1.524 (1.238–1.875)	** < 0.001**

OS overall survival, CI confidence interval, NA reference group or could not be evaluated. *Compared with each group (Log-Rank test or Omnibus test for univariate, Cox regression analysis with adjusted hazard for multivariate). *p* < 0.05 means statistically significant (highlighted in bold).

### Correlation analysis between MMP1 and tumor-immune microenvironment in HCC

**Figure 9:**
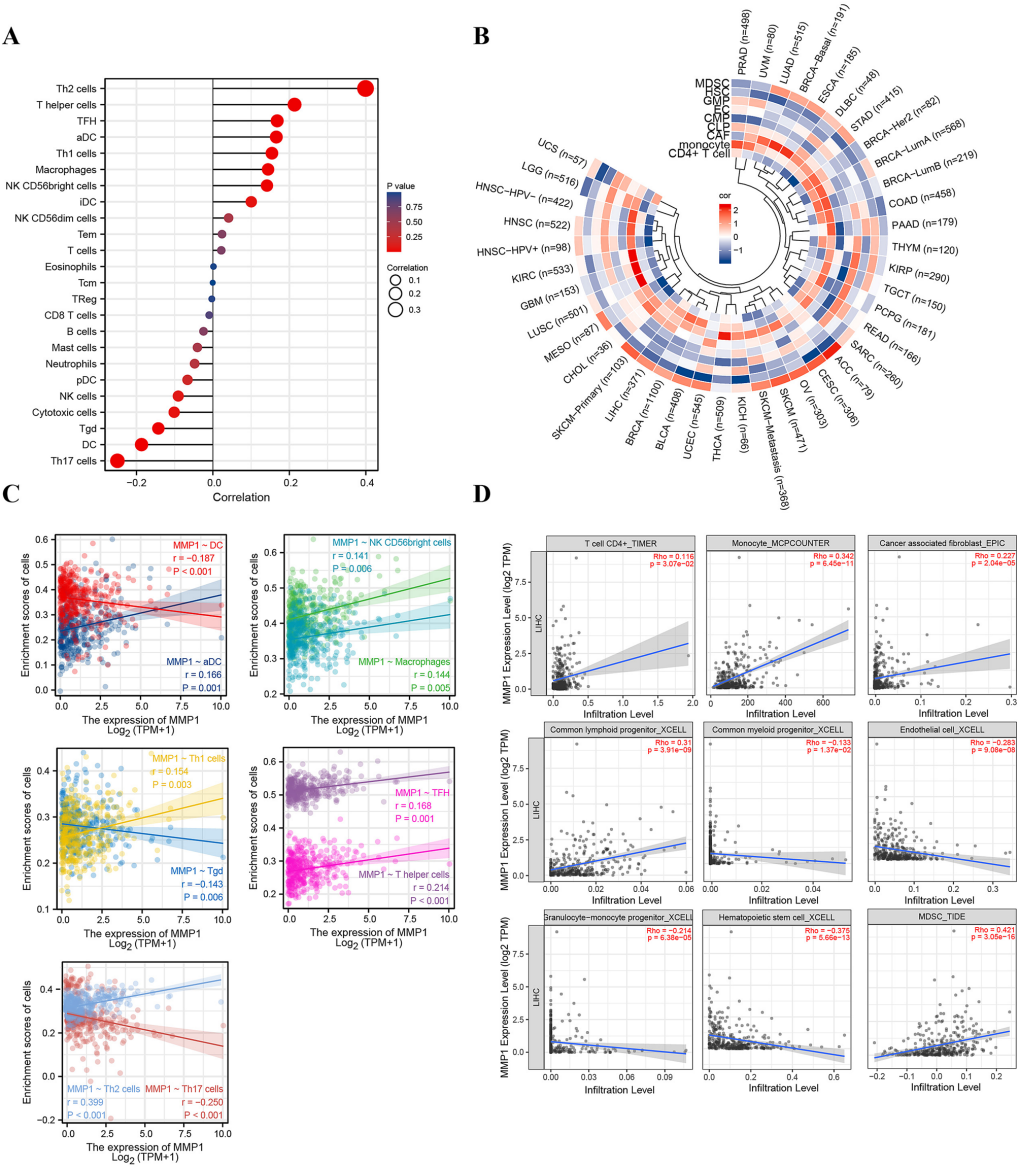
Multiple immune-related cells—MMP1 expression correlation analysis. (**A**) Lollipop plot of MMP1 expression and immune infiltration cells correlation in TCGA-LIHC; (**B**) Circle heatmap of MMP1 expression—immune-related factors correlation via TIMER2.0; (**C**) MMP1 expression and some types of immune-related cells; (**D**) Correlation analysis of other immune-related cells via TIMER2.0. *P < 0.05, **P < 0.01, ***P < 0.001.

Tumor infiltrating immunocytes, have an important role in the complex tumor-immune microenvironment, and have been shown to influence the progression of a variety of tumors. Thus, it was necessary for us to investigate any relationship between MMP1 expression and TIICs in HCC. 24 immune-related cells were first included into the correlation analysis (results in Fig. 9A). We used circle heatmap to perform other 9 immune-related cells as supplement via TIMER2.0 (Fig. 9B). Diverse of algorithms (i.e. XCELL, ssGSEA, MCPcounter, TIDE, EPIC, CIBERSORT, etc.) were applied for multi-verification (Fig.S2-S4, S5A). As shown in Fig. 9C and 9D, we found that a high MMP1 expression was negatively related with dendritic cell (DC) (r = -0.187, *P* < 0.001), T gamma delta (Tgd) (r =  0.143, *P* = 0.006), Th17 cells (r =  0.250, *P* < 0.001), common myeloid progenitor (CMP) (r =  0.133, *P* < 0.05), endothelial cells (EC) (r = -0.283, *P* < 0.001), granulocyte-monocyte progenitor (GMP) (r =  0.214, *P* < 0.001) and hematopoietic stem cell (r =  0.375, *P* < 0.001), while positively associated with activated DC (aDC) (r = 0.166, *P* = 0.001), NK CD56bright cells (r = 0.141, *P* = 0.006), macrophages (r = 0.144, *P* = 0.005), Th1 cells (r = 0.154, *P* = 0.003), T follicular helper (TFH) (r = 0.168, *P* = 0.001), T helper cells (r = 0.214, *P* < 0.001), Th2 cells (r = 0.399, *P* < 0.001), CD4^+^ T cells (r = 0.116, *P* < 0.05), monocytes (r = 0.342, *P* < 0.001), cancer associated fibroblast (CAF) (r = 0.227, *P* < 0.001), common lymphoid progenitor (CLP) (r = 0.31, *P* < 0.001) and myeloid-derived suppressor cells (MDSCs) (r = 0.421, *P* < 0.001).

Furthermore, survival analysis of covariates which comprised of MMP1 expression and several immune factors was performed. There were significant differences between each group of different gene expression and immune cell infiltration. Patients with MMP1 high expression + high macrophage/MDSC/T cell CD4 + infiltration (all *P* < 0.001) had a poorer OS (Fig. S5B).

**Figure 10:**
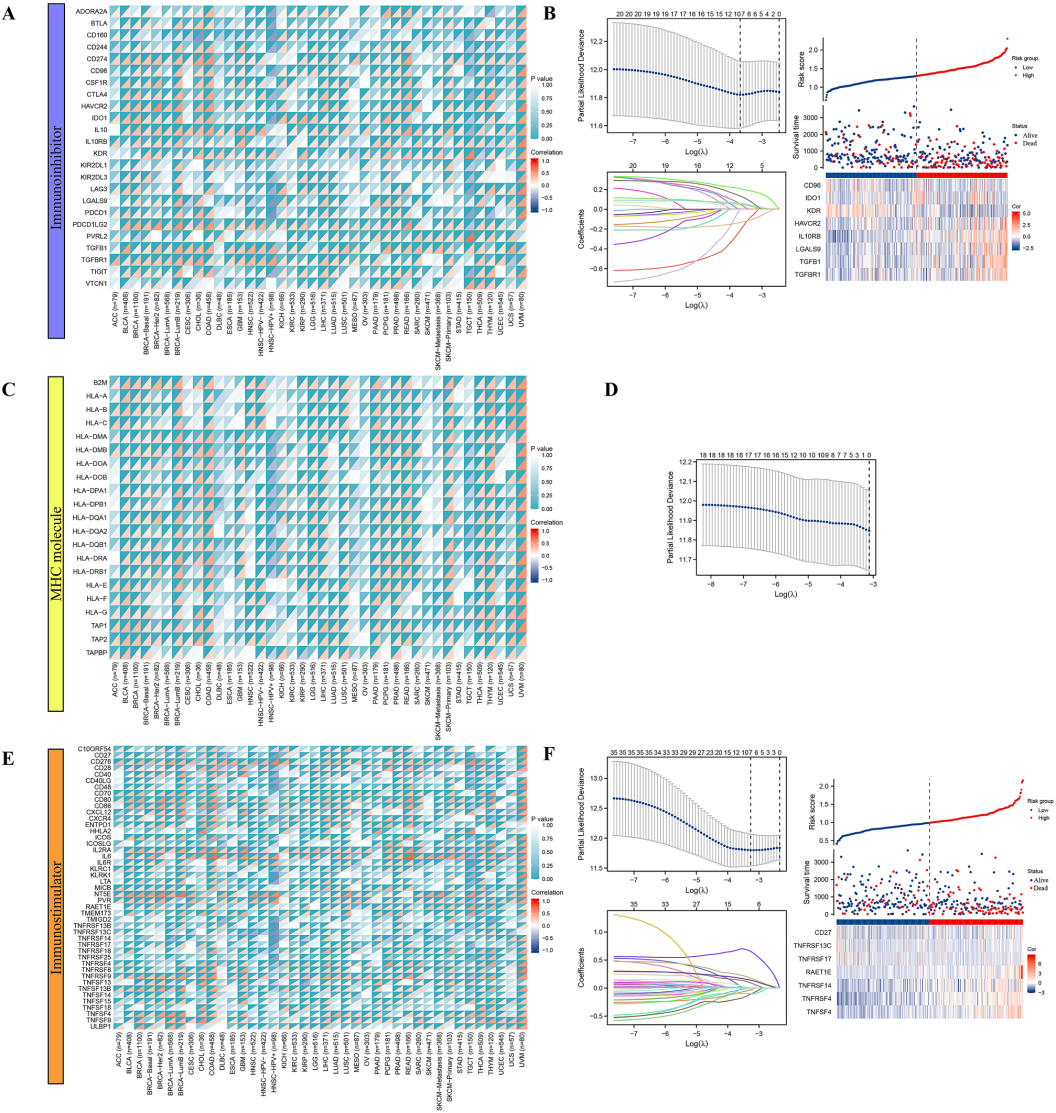
Analysis of correlation between MMP1 expression and multiple immune-related genes. (**A**) Immune-inhibitor genes—MMP1 expression heatmap; (**B**) Lasso analysis and risk factor prediction for immune suppressor genes; (**C**) MHC molecule genes—MMP1 expression correlation heatmap; (**D**) Lasso analysis for MHC molecules; (**E**) Immune-stimulator genes—MMP1 expression correlation heatmap; (**F**) Lasso analysis and risk factor prediction for immune-stimulating genes; MHC, major histocompatibility complex, *P < 0.05, **P < 0.01, ***P < 0.001.

In addition, we conducted correlation analysis between MMP1 and immunomodulators on all cancers of TCGA. We screened out the prominent risk factors correlated with MMP1 affecting HCC patient prognosis (Fig. 10). For immune-inhibitors, the potential correlation between MMP1 and immune-related genes was shown in a heatmap (Fig. 10A). We then conducted lasso regression analysis including the genes with *P* < 0.05 and screened 8 most correlated risk genes out. Patients in high-risk group might be associated with worse prognosis and positively with genes of HAVCR2, IL10RB, LGALS9, TGFB1 and TGFBR1, while negatively with gene of KDR (Fig. 10B). Although most of MHC molecules were significantly related with MMP1 (Fig. 10C), no risk factors predictive of prognosis were identified (Fig. 10D). In the case of immune-stimulators, correlation (Fig. 10E) and lasso regression (Fig. 10F) analyses were performed and 7 risk genes were screened out. Genes of RAET1E, TNFRSF14, TNFRSF4 and TNFSF4 seemed positively correlated with high-risk group accompanied worse prognosis, while CD27, TNFRSF13C and TNFRSF17 positively correlated with low-risk group.

### MMP1-related therapy

**Figure 11:**
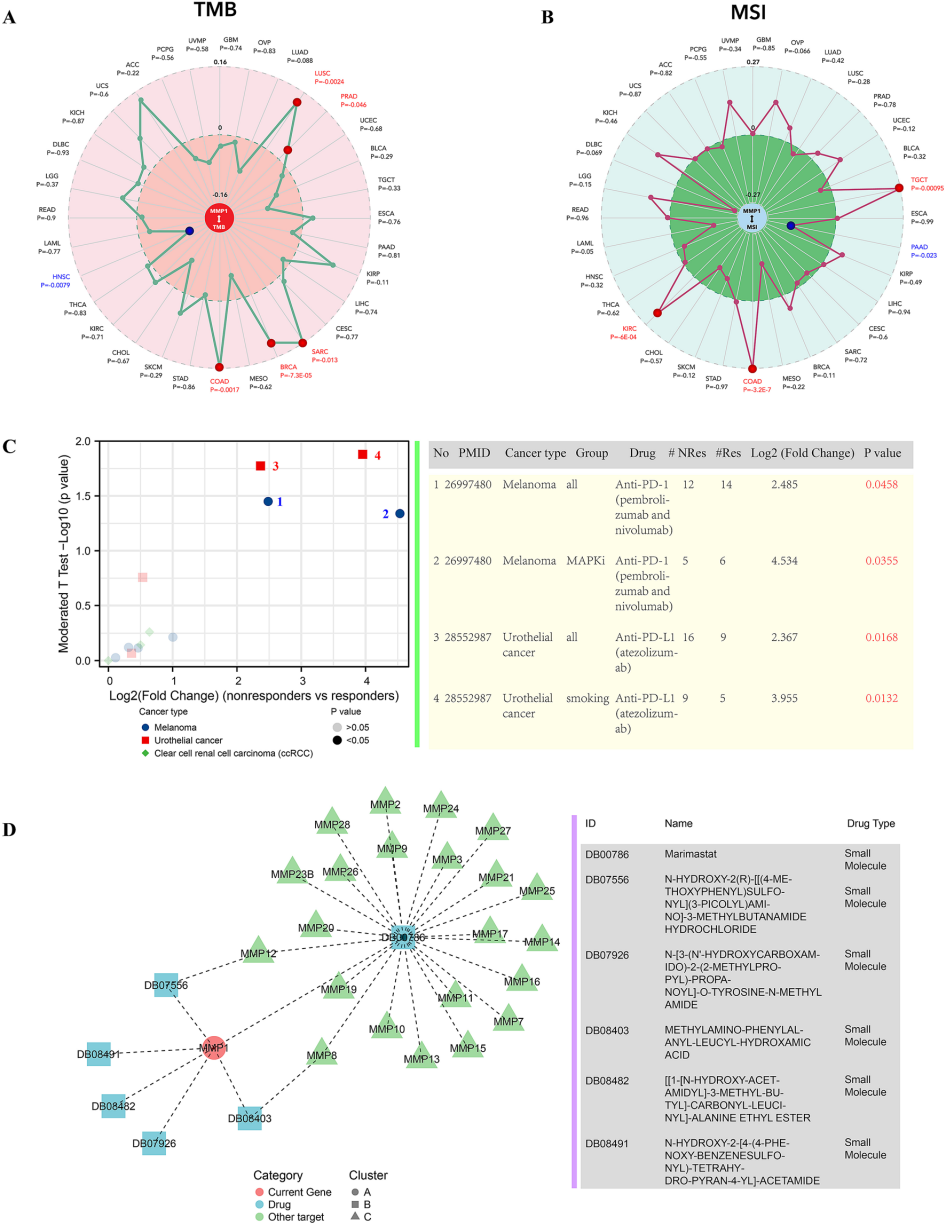
MMP1 immunotherapies and relevant drugs. (**A**) Correlation between MMP1 expression and tumor mutational burden (TMB); (**B**) Correlation between MMP1 expression and microsatellite instability (MSI); (**C**) Evaluation of immunotherapies associated with MMP1; (**D**) prediction of MMP1-related drugs. *P < 0.05, **P < 0.01, ***P < 0.001.

Studies have increasingly reported that TMB^65^ and MSI^66^ could be used as predictive biomarkers for cancer immunotherapy, which might be one of most popular methods to predict the therapeutic efficiency of immunotherapy on carcinomas. Therefore, we investigated the correlation between MMP1 expression and TMB/MSI in 32 types cancers via SangerBox. There was no significant correlation between MMP1 expression and TMB/MSI in HCC patients (Fig. 11A–B). Although response to MMP1-related immunotherapy was reported in melanoma (all group, *P* = 0.0458; MAPKi group, *P* = 0.0355) and urothelial cancer (all group, *P* = 0.0168; smoking group, *P* = 0.0132), its correlation or lack of in HCC has not been reported in any cohort studies thus far (Fig. 11C). As a supplement, we performed correlation analysis between MMP1 and IPS based on the cancer immunome (TCIA) database, which provides results of comprehensive immunogenomic analyses of next generation sequencing data (NGS) data for 20 solid cancers^67^. Although low-risk group had a higher score in patients with PD1-negtive and CTLA4-negtive (*P* < 0.05), there were no significant difference in PD1-positive, CTLA4-positive or both (Fig. S5C). For further exploration on MMP1-related drugs, we constructed a network diagram. Currently, drugs targeting MMP1 still remained in the experimental stage and Marimastat was the unique broad-spectrum MMPs inhibitor with oral activity (Fig. 11D).

## Discussion

Although the incidence and mortality of HCC have decreased in South-Eastern Asia due to hepatitis vaccination progress, it is still one of the leading causes of cancer related deaths world wide^1^. Lack of efficient prognostic factors leads to delayed diagnosis and intervention, which in turn considerably contribute to poor patient survival. In this study, we demonstrated that MMP1 expression was elevated in HCC and was significantly correlated with worse prognosis utilizing a bioinformatic analysis method based on public databases resources and experimental verification. Most importantly, we established an innovative MMP1-related prognostic model for predicting the survival probability of patients with HCC with good accuracy. The MMP1 expression had different relationships with corresponding TIICs and various immune-related genes in HCC. All the findings indicated an underlying mechanism of MMP1 expression in remodulating the tumor-immune microenvironment and immune escape. To our knowledge, our study is the first of a kind comprehensive analysis of MMP1 and establishes a novo prognostic model based on MMP1 expression in hepatocellular carcinoma, as well as evaluating MMP1-related risk factors of immunomodulators in HCC.

MMP1, as a member of MMPs, participates in the EMT which was identified as a strict programmed shift playing a crucial role in tumor invasion and metastasis^68^. MMPs could auto-activate and lead to a cascade of interaction activation between each other to enhance their influence in the EMT^20^. For invasive HCC, overexpression of MMP1 has been confirmed to correlate with an increased capacity of invasion and migration in HCC cells. The most likely mechanism involved is that of ECM degradation promoting the transmembrane migration of tumor cells^18^. This speculation combined with the BP of cell–cell adhesion via plasma-membrane adhesion molecules (Fig. 5C) would to some extent explain the outcomes of high expression of MMP1 in tumor tissues and poor prognosis in HCC patients. On the other hand, MMP1-mediated tumor progression could be regulated negatively by circDLC1^58^, CIC^59^ and MTAP^60^ and positively by 14–3-3σ^61^, MPP3^62^ and c-Jun^63^, indicating multiple pathological pathways of MMP1 carcinogenesis in HCC.

Although MMP1 expression has been reported to be high in malignant tumors and correlated with a poor prognosis in various cancers (ovarian, liver, lung, gastric, colorectal, and prostate), there is no comprehensive survival analysis between MMP1 expression and prognosis, nor a precise prognostic prediction model based on MMP1 in HCC. Consistent with previous studies, high expression of MMP1 is closely related with poor OS, PFS, DSS and PFI. However, high expression of MMP1 was not uniformly correlated with the poor OS for all clinicopathological characteristics. By performing the subgroup survival analysis, we found no significant correlation between MMP1 expression and T2–T4 stage, M1 stage, pathologic stage II-IV, R1&2 resection, AFP > 400, albumin < 3.5, prothrombin > 4, Child–Pugh B&C, vascular invasion, fibrosis ishak score 0–4 (all due to small sample size), tumor-free, gender of female and BMI > 25. Apart from tumor-free status, which had rare expression of MMP1, MMP1’s relationship with female gender and obesity needs further investigation.

Based on the univariate and multivariate analysis results, we suggest that monitoring MMP1 expression can be of significant value in early detection and mitigation of early HCC recurrence. Therefore, we developed a new predictive model that incorporates multiple clinical indications and MPP1 expression to predict patient prognosis. We validated the model using TCGA-LIHC and clinical HCC dataset from our center. Multi-center validation is still required.

According to resent studies, the integration of clinicopathological characteristics and TIICs can be a clinical predictive model for the efficiency of immunotherapy^26,69^. The genesis and development of tumors can involve large numbers of immune infiltrating cells and inflammatory mediators. Although MMP1 is involved in the tumor-immune-related progression of some carcinomas, there is barely any studies regarding the interaction between MMP1 and TIICs in HCC proliferation and migration. Our study, presents relationship between MMP1 expression and different TIICs or immunomodulators, signifying a close connection between MMP1 and immune infiltration in HCC patients. Although the function of TIICs in carcinogenesis is still controversial, a cluster of studies have reported that MMP1 alongside TIICs plays a vital role in tumor progression^70–72^. To further investigate the relationship between MMP1 expression and immune-related cells infiltration in HCC, we analyzed the data using the spearman test, ssGESA and other statistical algorithms (Figs. 9, S2-4, S5A). As reported in prior studies, high MMP1 expression may promote the production of tumor-killing immune cells (aDC, NK CD56bright cells, macrophages, Th1/2, TFH, T helper cells and CD4^+^ T cells) and cause regional inflammation and fibrosis (monocyte and CAF). The positive correlation between MMP1 and MDSC could be probably explained by MDSC suppression of ability of immune cells to respond thus leading to tumor progression. In the same way, the negative correlation between MMP1 and DC/GMP may be a result of the depletion caused by continuous activation of DC and monocyte. The poor survival associated with high MMP1 expression + high macrophage/MDSC/T cell CD4 + infiltration also indicated that these risk factors might contribute to tumor progression synergistically. However, it is difficult to explain the negative correlation between MMP1 and Th17 (induce immune response to bacteria and fungi), Tgd (adjuvant tumor killing), EC, CMP and HSC, as well as the positive correlation with CLP. Tumor-immune microenvironment is extremely complex and involved with a multitude of undisclosed mechanisms. All the correlations above need to be validation and explored deeper. The results of TMB/MSI evaluation suggested the patients with HCC might not readily benefit from the treatment of PD-1, necessitating the exploration of inhibitors targeted to new immunosuppressive site. In consistent with it, the results of IPS correlation analysis did not reveal more available immunotherapeutic information for LIHC patients yet. Hence, one or more HCC-related immunotherapy cohorts are vital for future research.

Understanding mechanism of the tumor-immune microenvironment has been at the frontier of research to screen out related genes that can serve as biomarkers for diagnosis and prognosis or therapeutic targets^73^. Using lasso regression analysis, we screened out several potential immune-related genes with significant correlation with MMP1.

Although we conducted numerous analyses, there were several notable limitations to our study. First, since we used many of databases and statistic methods in attempting to elaborate the role of MMP1 on tumorigenesis and prognosis in HCC, cask effect appeared when harmonizing the data. This is mainly a result of data updates being out of sync or the source having a single function. We conducted the experiments and analyzed the clinical data to provide a more concrete basis for the conclusions, but did not conduct the experiments for immune-related cells/genes since a large amount of data is required to complete the experimentation and such experimentation is not feasible at a single center. The correlation between MMP1 and TIICs was detectable but not strong, and some contradictory results were difficult to explain. This is an important direction and terra incognita for further research. Since this was a bioinformatic analysis, the batch effect of samples and cross-platforms, the difference in the sample sizes, types and data processing methods among various databases were common and difficult to eliminate. Although we had unified data cleaning and batch effect correction, the batch effect of cross-platforms may still exist.

The internal validation of the MMP1 based nomogram relied on a relative small dataset, therefore external validation with larger sample sizes is required.

In conclusion, our study revealed a close relationship between high MMP1 expression and poor prognosis in HCC and MMP1 involvement in tumor-immune cell infiltration and immunomodulators. We also suggest a model to predict prognosis in HCC patient with good accuracy. Although some mechanisms associated with MMP1 are unknown, we still have reason to believe that MMP1 is a promising prospective prognostic biomarker in HCC. The potential mechanism of MMP1 and tumor immune microenvironment and relevant immunotherapy cohort can be the focus of future research.

## Supplementary Information

Supplementary Information 1.

Supplementary Information 2.

Supplementary Information 3.

Supplementary Information 4.

Supplementary Information 5.

Supplementary Information 6.

Supplementary Information 7.

## Data Availability

All data generated or analyzed during this study are included in this published article [and its supplementary information files]. Links and illustrations are as follows: (1) TCGA-LIHC: https://portal.gdc.cancer.gov/ (Cleaning edition of LIHC data in supplementary materials, labelled “LIHC rnaseq clinical raw”). (2) GSE14520: https://www.ncbi.nlm.nih.gov/geo/query/acc.cgi (Cleaning edition of GSE14520 data in supplementary materials, labelled “GSE14520”). (3) GSE25097: https://www.ncbi.nlm.nih.gov/geo/query/acc.cgi?acc=GSE25097 (Cleaning edition of GSE25097 data in supplementary materials, labelled “GSE25097”). (4) Clinical data of 108 samples were available in supplementary materials (labelled “clinical baseline” and “clinical Cox regression”).
