# Supplementary material for: Comprehensive bioinformatic analysis of MMP1 in hepatocellular carcinoma and establishment of relevant prognostic model
Source: Sci Rep. 2022 Aug 10;12:13639. doi: 10.1038/s41598-022-17954-x (PMC9365786; doi:10.1038/s41598-022-17954-x)

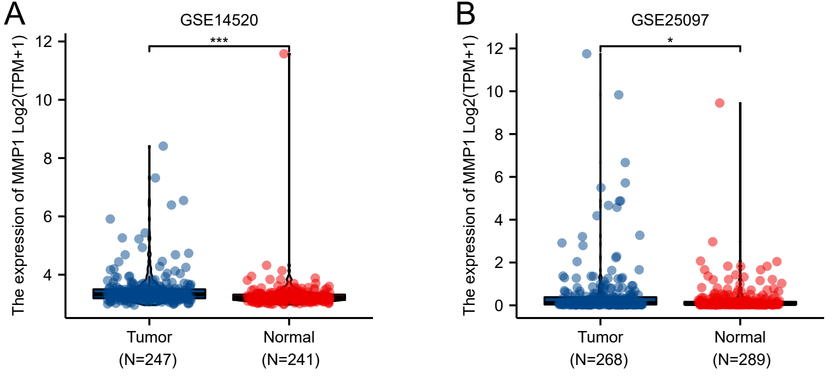

A
GSE14520
***
The expression of MMP1 Log2(TPM+1)
12
10
8
6
4
Tumor
(N=247)
Normal
(N=241)
B
GSE25097
*
The expression of MMP1 Log2(TPM+1)
12
10
8
6
4
2
0
Tumor
(N=268)
Normal
(N=289)

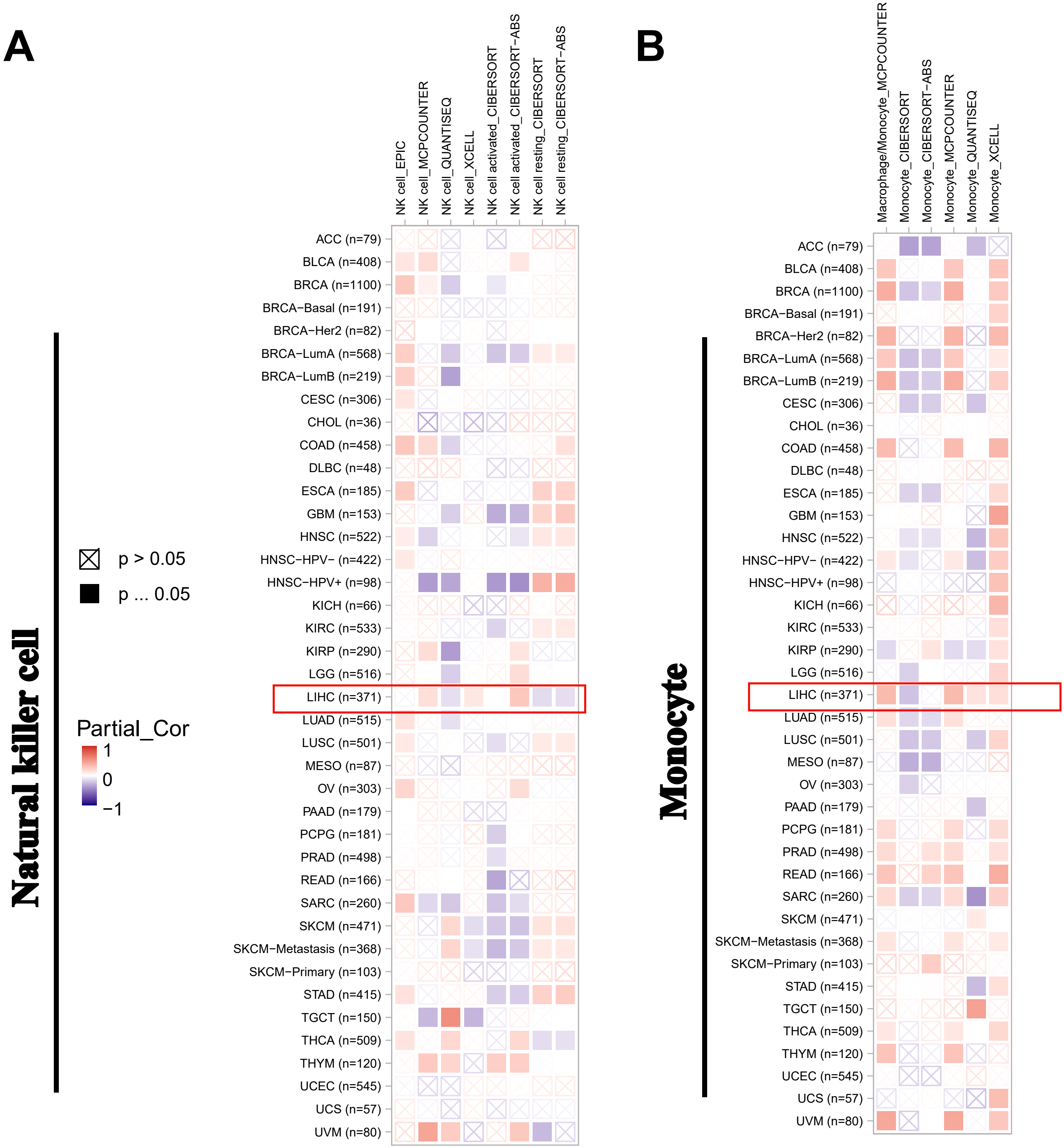

A
B
NK cell_EPIC
NK cell_MCPCOUNTER
NK cell_QUANTISEQ
NK cell_XCELL
NK cell activated_CIBERSORT
NK cell activated_CIBERSORT−ABS
NK cell resting_CIBERSORT
NK cell resting_CIBERSORT−ABS
Macrophage/Monocyte_MCPCOUNTER
Monocyte_CIBERSORT
Monocyte_CIBERSORT−ABS
Monocyte_MCPCOUNTER
Monocyte_QUANTISEQ
Monocyte_XCELL
ACC (n=79)
BLCA (n=408)
BRCA (n=1100)
BRCA−Basal (n=191)
BRCA−Her2 (n=82)
BRCA−LumA (n=568)
BRCA−LumB (n=219)
CESC (n=306)
CHOL (n=36)
COAD (n=458)
DLBC (n=48)
ESCA (n=185)
GBM (n=153)
HNSC (n=522)
HNSC−HPV− (n=422)
HNSC−HPV+ (n=98)
KICH (n=66)
KIRC (n=533)
KIRP (n=290)
LGG (n=516)
LIHC (n=371)
LUAD (n=515)
LUSC (n=501)
MESO (n=87)
OV (n=303)
PAAD (n=179)
PCPG (n=181)
PRAD (n=498)
READ (n=166)
SARC (n=260)
SKCM (n=471)
SKCM−Metastasis (n=368)
SKCM−Primary (n=103)
STAD (n=415)
TGCT (n=150)
THCA (n=509)
THYM (n=120)
UCEC (n=545)
UCS (n=57)
UVM (n=80)
p > 0.05
p ... 0.05
Partial_Cor
1
0
−1
Natural killer cell
Monocyte

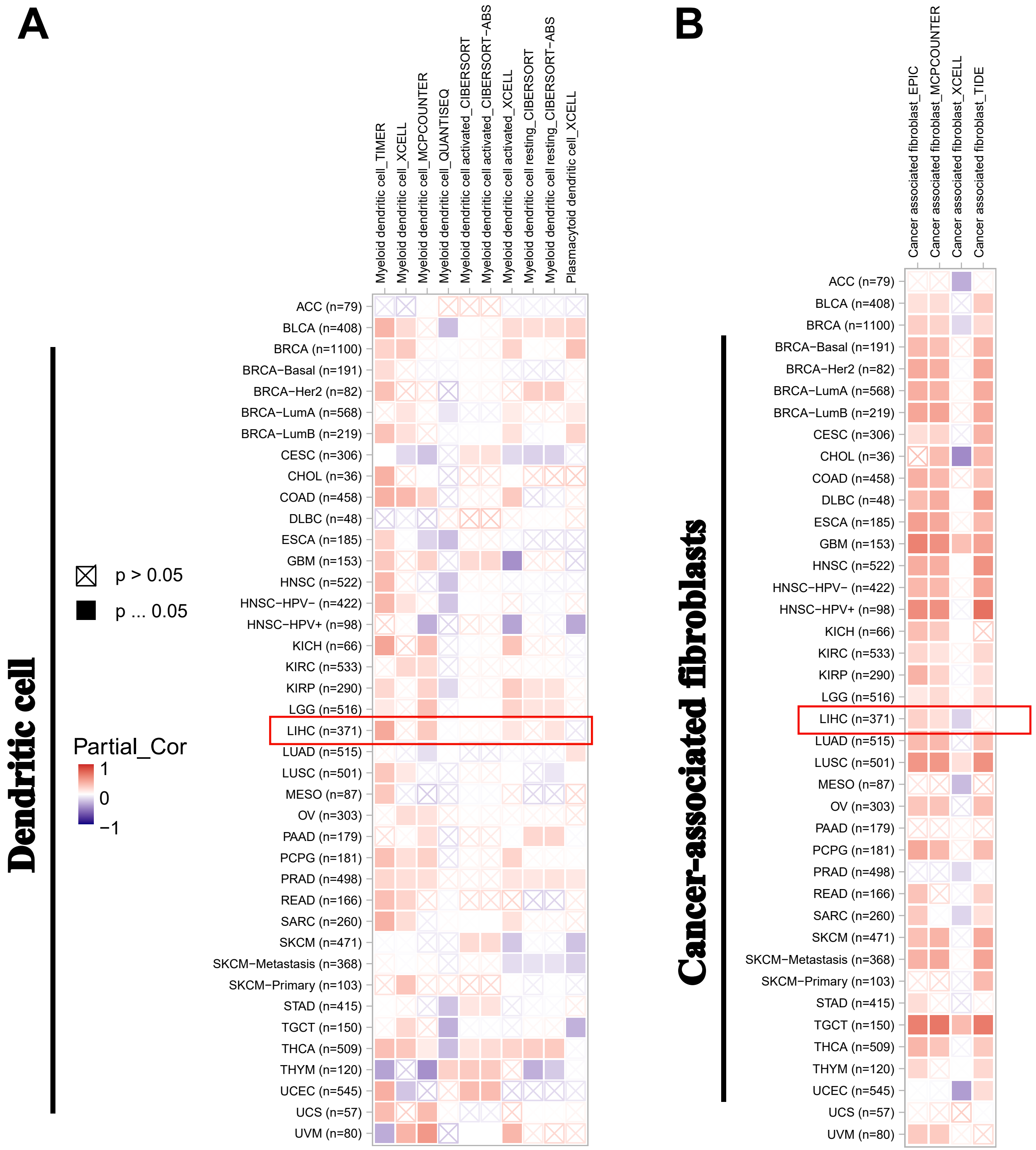

A
B
Myeloid dendritic cell_TIMER
Myeloid dendritic cell_XCELL
Myeloid dendritic cell_MCPCOUNTER
Myeloid dendritic cell_QUANTISEQ
Myeloid dendritic cell activated_CIBERSORT
Myeloid dendritic cell activated_CIBERSORT−ABS
Myeloid dendritic cell activated_XCELL
Myeloid dendritic cell resting_CIBERSORT
Myeloid dendritic cell resting_CIBERSORT−ABS
Plasmacytoid dendritic cell_XCELL
Cancer associated fibroblast_EPIC
Cancer associated fibroblast_MCPCOUNTER
Cancer associated fibroblast_XCELL
Cancer associated fibroblast_TIDE
ACC (n=79)
BLCA (n=408)
BRCA (n=1100)
BRCA−Basal (n=191)
BRCA−Her2 (n=82)
BRCA−LumA (n=568)
BRCA−LumB (n=219)
CESC (n=306)
CHOL (n=36)
COAD (n=458)
DLBC (n=48)
ESCA (n=185)
GBM (n=153)
HNSC (n=522)
HNSC−HPV− (n=422)
HNSC−HPV+ (n=98)
KICH (n=66)
KIRC (n=533)
KIRP (n=290)
LGG (n=516)
LIHC (n=371)
LUAD (n=515)
LUSC (n=501)
MESO (n=87)
OV (n=303)
PAAD (n=179)
PCPG (n=181)
PRAD (n=498)
READ (n=166)
SARC (n=260)
SKCM (n=471)
SKCM−Metastasis (n=368)
SKCM−Primary (n=103)
STAD (n=415)
TGCT (n=150)
THCA (n=509)
THYM (n=120)
UCEC (n=545)
UCS (n=57)
UVM (n=80)
p > 0.05
p ... 0.05
Partial_Cor
1
0
−1
Dendritic cell
Cancer-associated fibroblasts

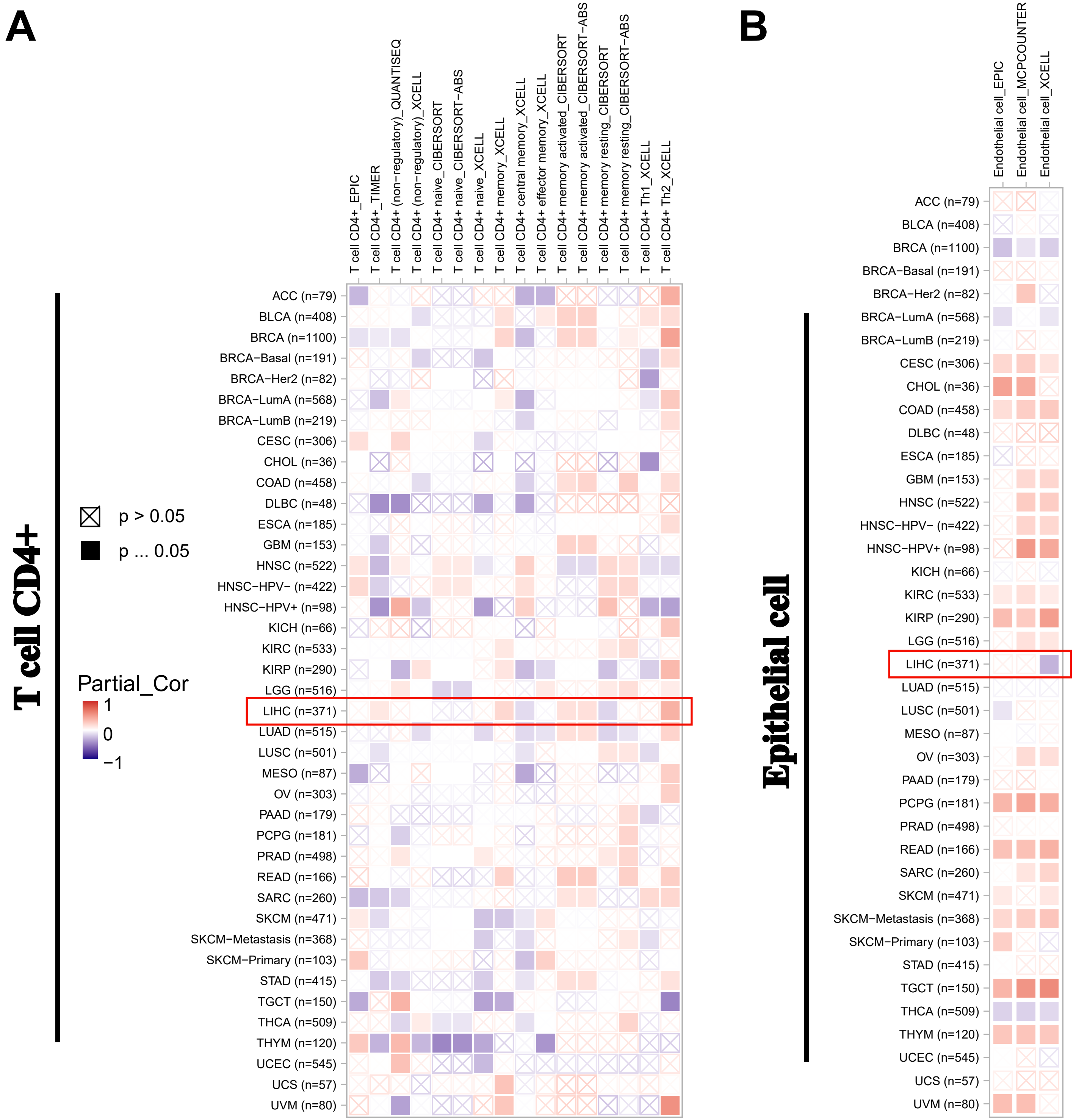
A
B
T cell CD4+
Epithelial cell
p > 0.05
p ... 0.05
Partial_Cor
1
0
−1
T cell CD4+_EPIC
T cell CD4+_TIMER
T cell CD4+ (non−regulatory)_QUANTISEQ
T cell CD4+ (non−regulatory)_XCELL
T cell CD4+ naive_CIBERSORT
T cell CD4+ naive_CIBERSORT−ABS
T cell CD4+ naive_XCELL
T cell CD4+ memory_XCELL
T cell CD4+ central memory_XCELL
T cell CD4+ effector memory_XCELL
T cell CD4+ memory activated_CIBERSORT
T cell CD4+ memory activated_CIBERSORT−ABS
T cell CD4+ memory resting_CIBERSORT
T cell CD4+ memory resting_CIBERSORT−ABS
T cell CD4+ Th1_XCELL
T cell CD4+ Th2_XCELL
Endothelial cell_EPIC
Endothelial cell_MCPCOUNTER
Endothelial cell_XCELL
ACC (n=79)
BLCA (n=408)
BRCA (n=1100)
BRCA−Basal (n=191)
BRCA−Her2 (n=82)
BRCA−LumA (n=568)
BRCA−LumB (n=219)
CESC (n=306)
CHOL (n=36)
COAD (n=458)
DLBC (n=48)
ESCA (n=185)
GBM (n=153)
HNSC (n=522)
HNSC−HPV− (n=422)
HNSC−HPV+ (n=98)
KICH (n=66)
KIRC (n=533)
KIRP (n=290)
LGG (n=516)
LIHC (n=371)
LUAD (n=515)
LUSC (n=501)
MESO (n=87)
OV (n=303)
PAAD (n=179)
PCPG (n=181)
PRAD (n=498)
READ (n=166)
SARC (n=260)
SKCM (n=471)
SKCM−Metastasis (n=368)
SKCM−Primary (n=103)
STAD (n=415)
TGCT (n=150)
THCA (n=509)
THYM (n=120)
UCEC (n=545)
UCS (n=57)
UVM (n=80)

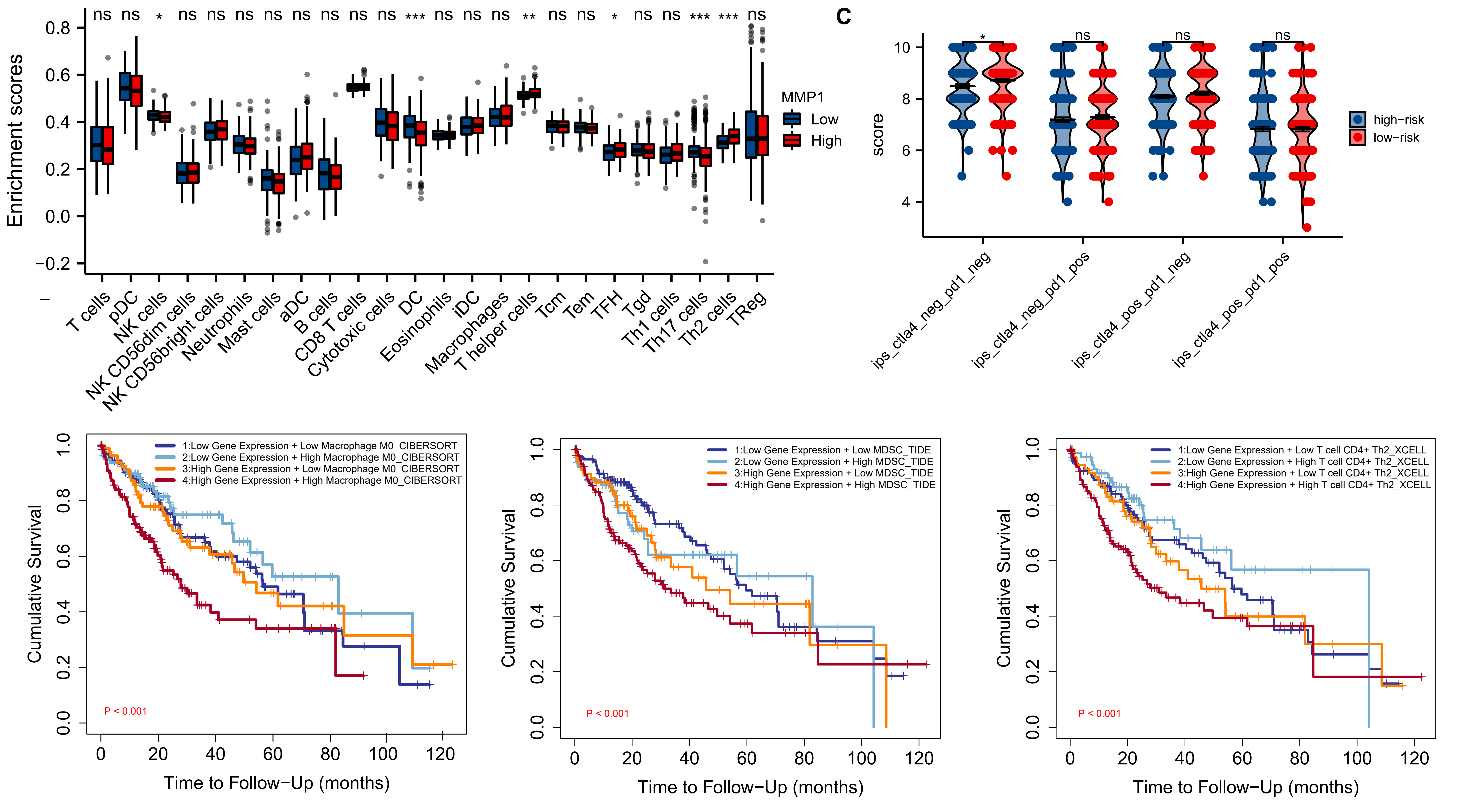

ns ns * ns ns ns ns ns ns ns ns *** ns ns ns ** ns ns * ns ns *** *** ns
Enrichment scores
0.8
0.6
0.4
0.2
0.0
−0.2
MMP1
Low
High
T cells
pDC
NK cells
NK CD56dim cells
NK CD56bright cells
Neutrophils
Mast cells
aDC
B cells
CD8 T cells
Cytotoxic cells
DC
Eosinophils
iDC
Macrophages
T helper cells
Tcm
Tem
TFH
Tgd
Th1 cells
Th17 cells
Th2 cells
TReg
C
score
10
8
6
4
* ns ns ns
high−risk
low−risk
ips_ctla4_neg_pd1_neg
ips_ctla4_neg_pd1_pos
ips_ctla4_pos_pd1_neg
ips_ctla4_pos_pd1_pos
Cumulative Survival
Time to Follow−Up (months)
1:Low Gene Expression + Low Macrophage M0_CIBERSORT
2:Low Gene Expression + High Macrophage M0_CIBERSORT
3:High Gene Expression + Low Macrophage M0_CIBERSORT
4:High Gene Expression + High Macrophage M0_CIBERSORT
P < 0.001
1:Low Gene Expression + Low MDSC_TIDE
2:Low Gene Expression + High MDSC_TIDE
3:High Gene Expression + Low MDSC_TIDE
4:High Gene Expression + High MDSC_TIDE
P < 0.001
1:Low Gene Expression + Low T cell CD4+ Th2_XCELL
2:Low Gene Expression + High T cell CD4+ Th2_XCELL
3:High Gene Expression + Low T cell CD4+ Th2_XCELL
4:High Gene Expression + High T cell CD4+ Th2_XCELL
P < 0.001
0.0 0.2 0.4 0.6 0.8 1.0
0 20 40 60 80 100 120

Fig.S6 GAPDH group(1,3)

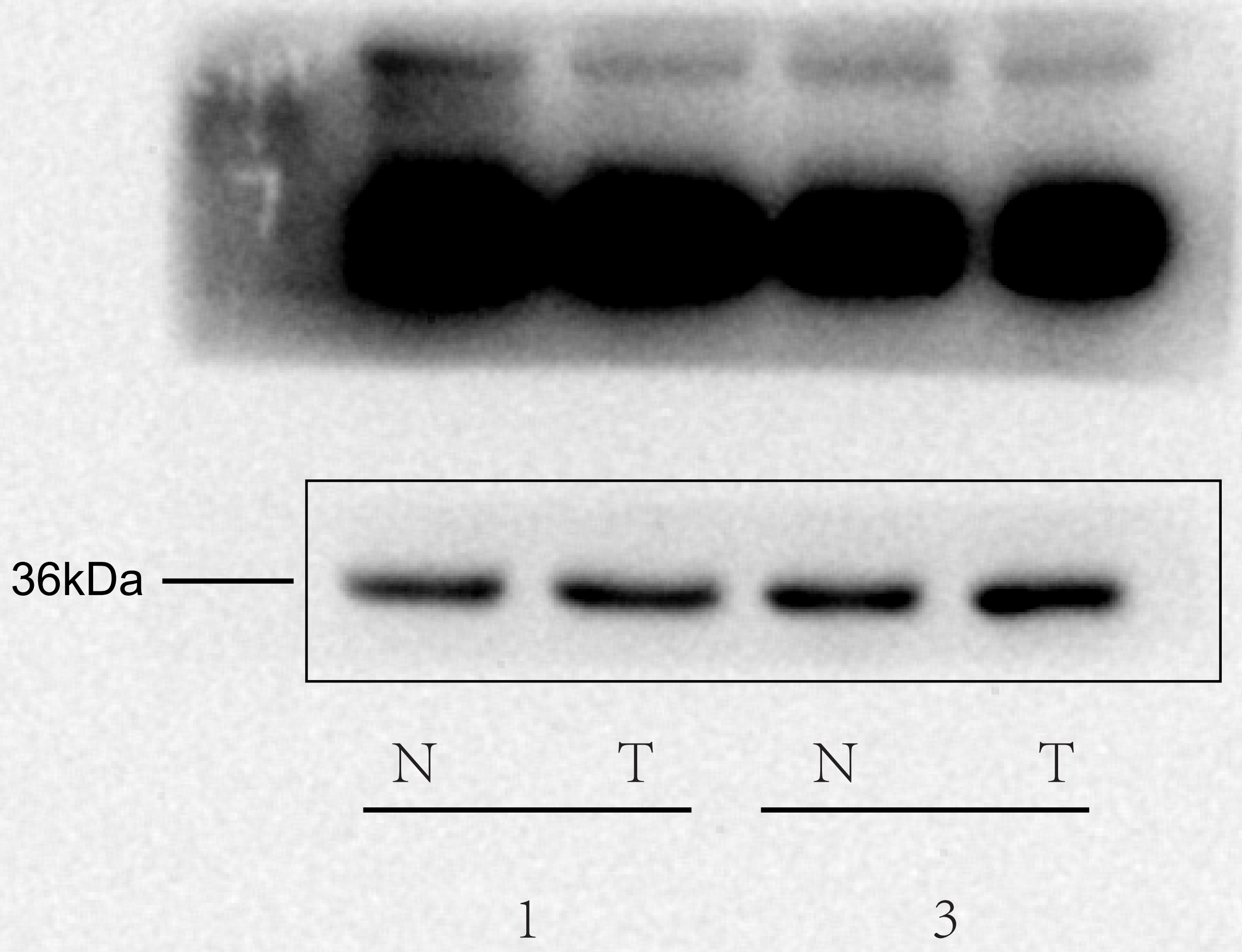

Fig.S7 MMP1group (1,2)

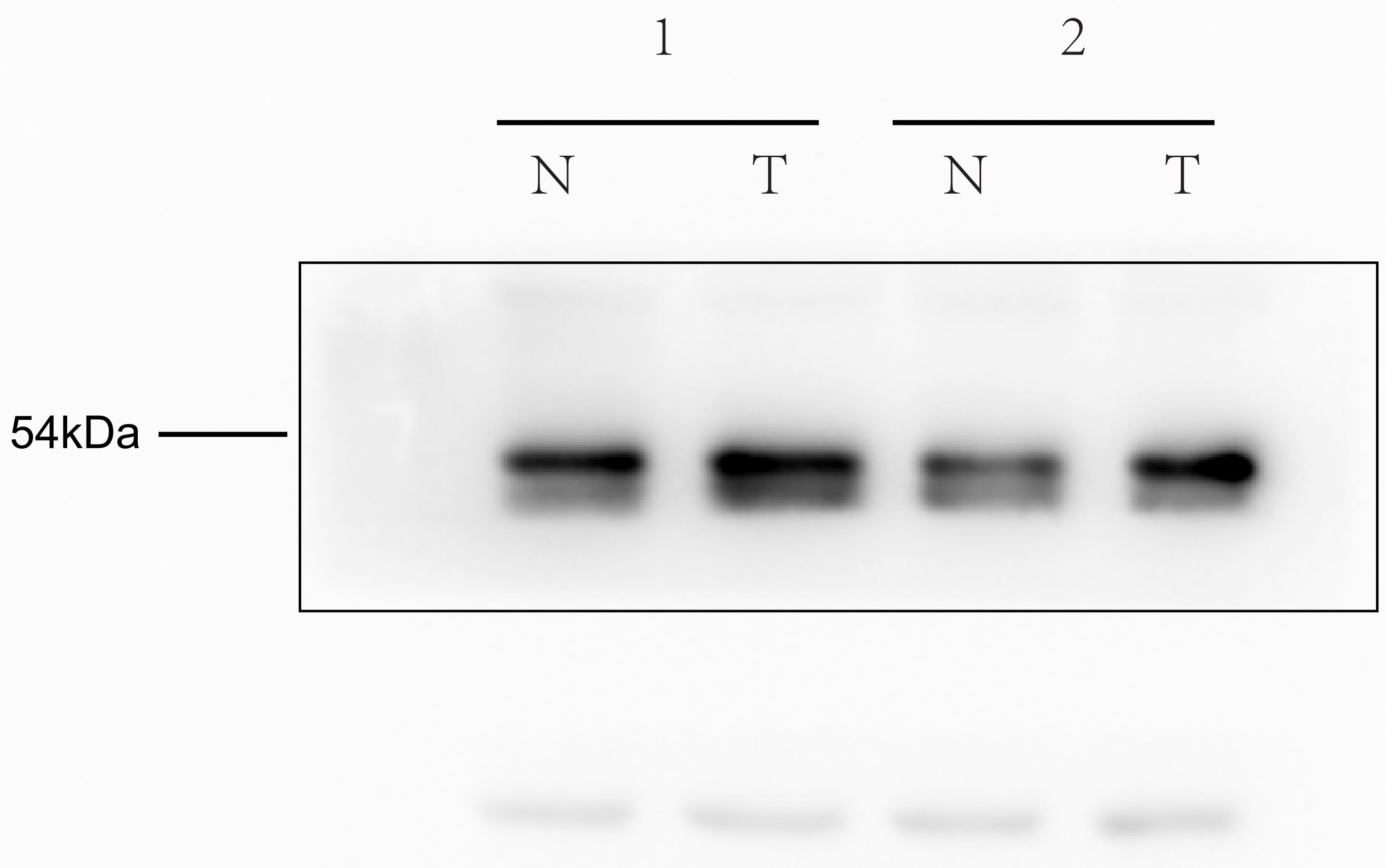

Fig.S8 MMP1group(3)+GAPDH group(2)

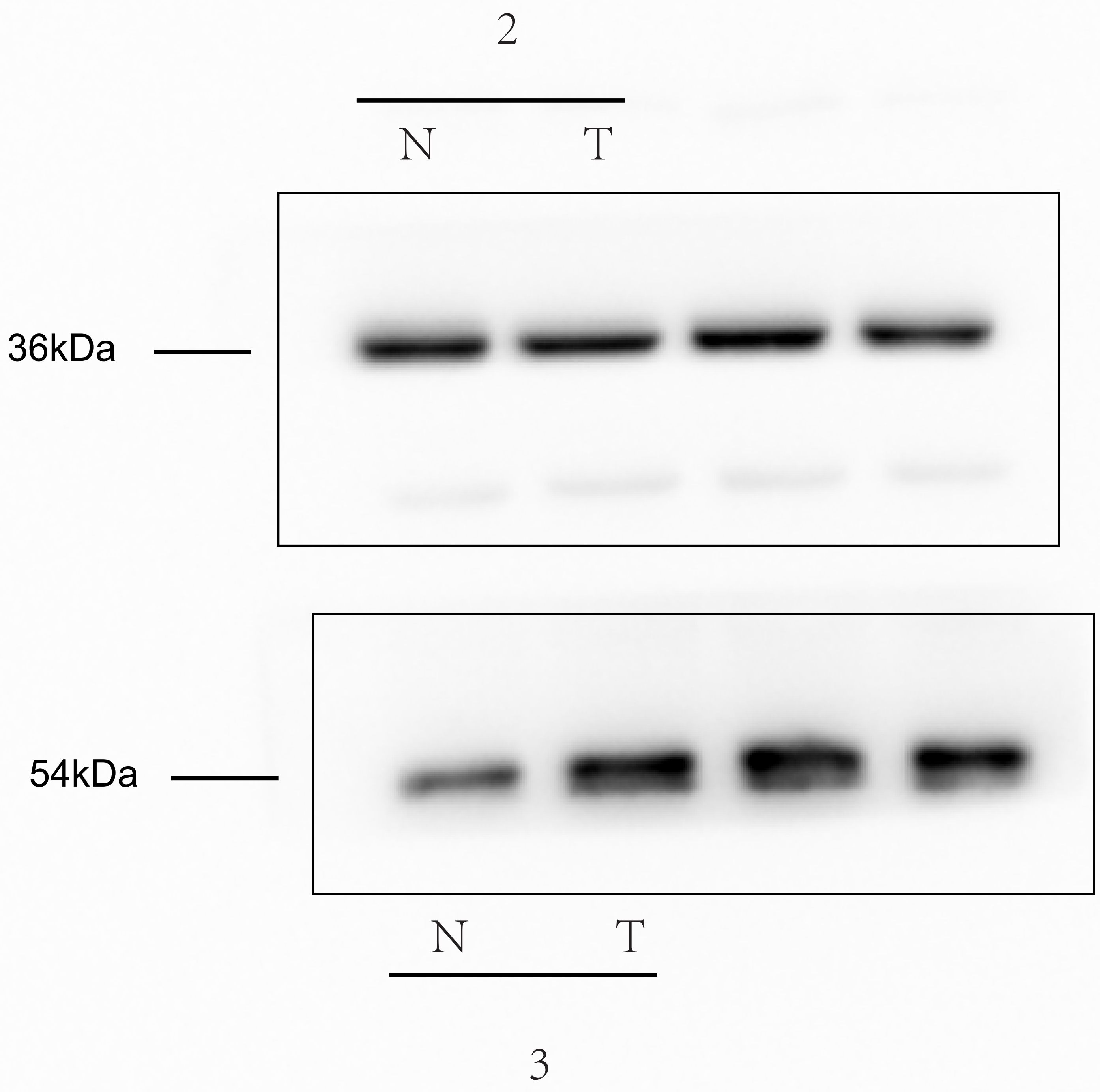

# Fig.S9 GAPDH group(1,3) low exposure

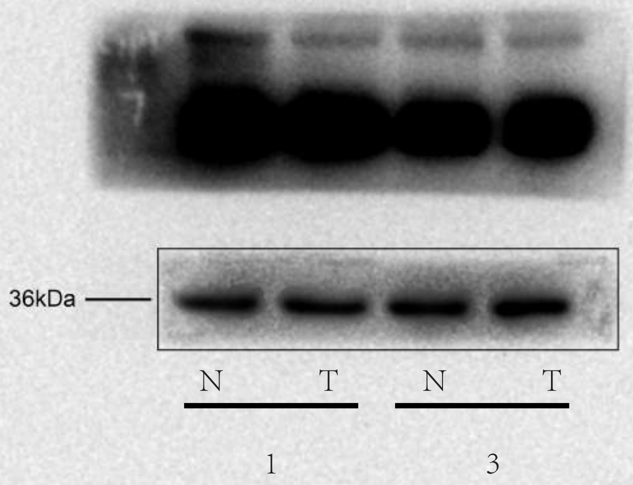

Fig.S10 MMP1group (1,2) low exposure

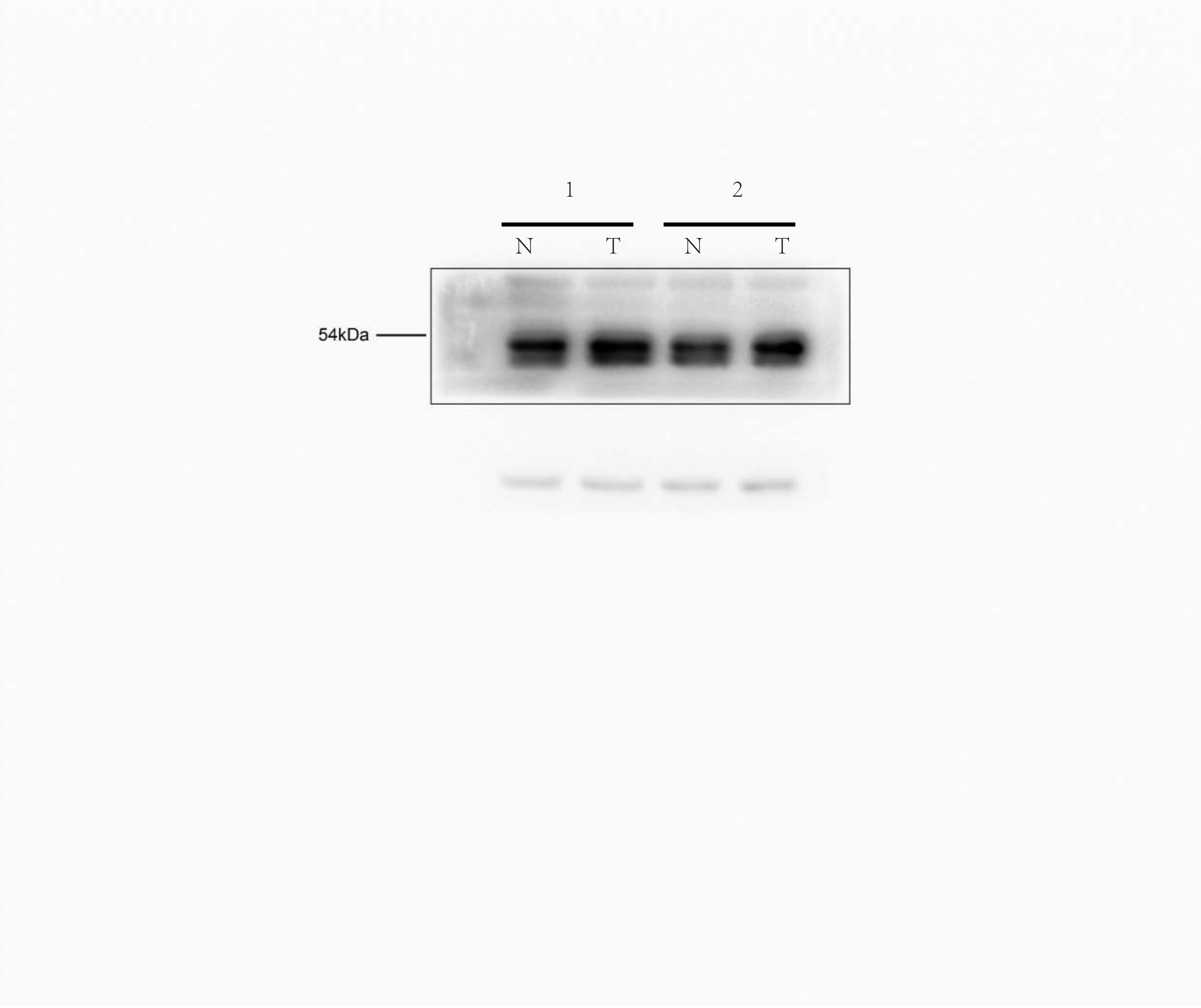

# Fig.S11 MMP1group(3)+GAPDH group(2) low exposure

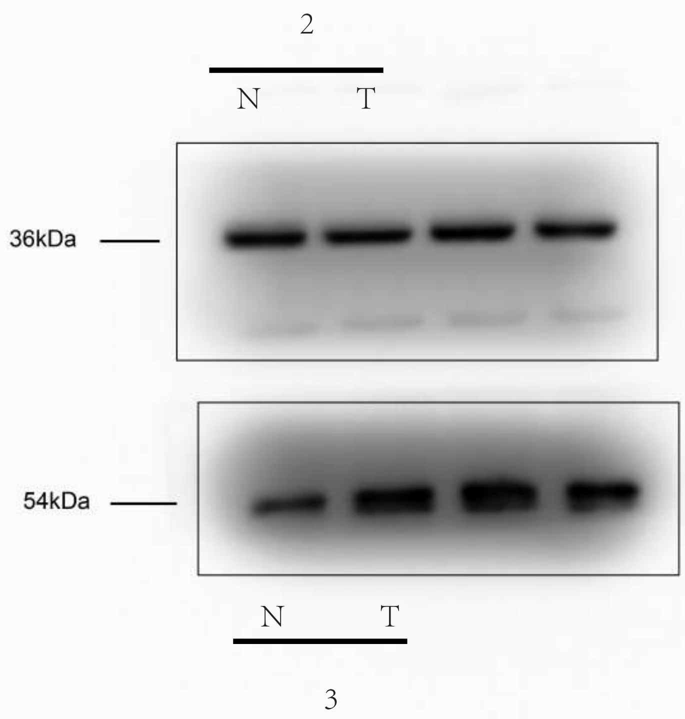

Supplement: Supplementary file 3 — Supplementary Information 3. [file 41598_2022_17954_MOESM3_ESM.pdf]
