# Supplementary material for: Comprehensive bioinformatic analysis of MMP1 in hepatocellular carcinoma and establishment of relevant prognostic model
Source: Sci Rep. 2022 Aug 10;12:13639. doi: 10.1038/s41598-022-17954-x (PMC9365786; doi:10.1038/s41598-022-17954-x)
Supplement: Supplementary file 7 — Supplementary Information 7. [file 41598_2022_17954_MOESM7_ESM.docx]

# Supplementary figure legends

**Figure S1. MMP1 expression levels validation analysis.**

(A) Analysis of MMP1 expression level in LIHC based on GSE14520; (B) Analysis of MMP1 expression level in LIHC based on GSE25097. **P* < 0.05, ***P* < 0.01, ****P* < 0.001.

**Figure S2. Correlation analysis between MMP1 and NK cell/Monocyte.**

(A) Heatmap of MMP1 expression and NK cell among pan-cancer with different algorithms; (B) Heatmap of MMP1 expression and Monocyte among pan-cancer with different algorithms. NK, natural killer; **P* < 0.05, ***P* < 0.01, ****P* < 0.001.

**Figure S3. Correlation analysis between MMP1 and DC/CAF.**

(A) Heatmap of MMP1 expression and DC among pan-cancer with different algorithms; (B) Heatmap of MMP1 expression and CAF among pan-cancer with different algorithms. DC, dendritic cell; CAF, cancer-associated fibroblasts; **P* < 0.05, ***P* < 0.01, ****P* < 0.001.

**Figure S4. Correlation analysis between MMP1 and T cell CD4+/EC.**

(A) Heatmap of MMP1 expression and T cell CD4+ among pan-cancer with different algorithms; (B) Heatmap of MMP1 expression and EC among pan-cancer with different algorithms. EC, epithelial cell; **P* < 0.05, ***P* < 0.01, ****P* < 0.001.

**Figure S5. Relevant analysis of immune infiltration, clinical outcome and IPS.**

(A) Correlation analysis of MMP1 and 23 immune cells using ssGESA algorithm; (B) Survival analysis of MMP1 and several immune cells synergistically; (C) Correlation analysis of MMP1 and IPS in LIHC. IPS, immunophenotyping score; **P* < 0.05, ***P* < 0.01, ****P* < 0.001.

**Figure S6. Original images of GAPDH blots (group1 and group3)**

**Figure S7 Original images of MMP1 blots (group1 and group2)**

**Figure S8. Original images of GAPDH blots (group2) and MMP1 blots (group3)**

**Figure S9. Original images of GAPDH blots (group1 and group3) low exposure**

**Figure S10 Original images of MMP1 blots (group1 and group2) low exposure**

**Figure S11. Original images of GAPDH blots (group2) and MMP1 blots (group3) low exposure**
